# *FvGI–FvCO–FvFT1* pathway activates *FvTFL1* in long days to repress flowering in woodland strawberry (*Fragaria vesca*)

**DOI:** 10.1111/nph.71555

**Published:** 2026-08-28

**Authors:** Quan Zhou, Sergei Lembinen, Tuomas Toivainen, Takeshi Kurokura, Guangxun Fan, Paula Elomaa, Elli Koskela, Timo Hytönen

**Affiliations:** ^1^ Department of Agricultural Sciences, Viikki Plant Science Centre University of Helsinki 00790 Helsinki Finland; ^2^ Faculty of Agriculture Utsunomiya University Mine 350 Utsunomiya Tochigi 321‐8505 Japan

**Keywords:** adaptation, epistasis, flowering, *Fragaria vesca*, photoperiod, woodland strawberry

## Abstract

- Photoperiod is a stable seasonal signal. Although photoperiodic flowering is well understood in short‐day (SD) and long‐day (LD) annual plants, the regulatory mechanisms in perennials remain elusive. In a perennial woodland strawberry (*Fragaria vesca*), flowering is induced in SDs in autumn and plants flower following spring, while in plants with mutated *FvTERMINAL FLOWER1* (*FvTFL1*), LDs induce flowering.
- We investigated photoperiodic flowering of *F. vesca* through phenotypic and molecular characterization of transgenic lines and their crosses. We studied natural variation in flowering time and gene expression in European accessions and explored their correlations with climatic, geographical and genetic origins.
- We showed that *FvGIGANTEA* (*FvGI*) activates *FvFLOWERING LOCUS T1* (*FvFT1*) via *FvCONSTANS* (*FvCO*) in LDs resulting in early flowering in *fvtfl1* mutant. In natural accessions, FvFT1 activates *FvTFL1* to repress flowering, and decreasing expression of *FvFT1* and *FvTFL1* towards colder climates in the east and north correlates with earlier flowering.
- We define a photoperiodic flowering pathway controlling floral transition of perennial *F. vesca* in autumn that differs from known mechanisms in annual and perennial plants. Our findings open new avenues to understand how perennial plants cope with changing seasons across climatic and geographical ranges.

## Graphical abstract

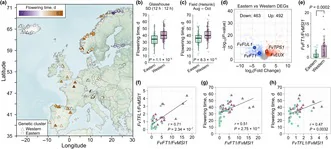

## Introduction

Perennials comprise the largest fraction of plant species on Earth with an increasing proportion towards higher latitudes (Poppenwimer *et al*., 2023). They are mostly polycarpic plants that repeat yearly cycles of vegetative and generative growth to ensure successful reproduction during their lifespan, in contrast to monocarpic annual and biennial species that rely on a single flowering event (Albani & Coupland, 2010). A common perennial life cycle comprises the formation of overwintering floral buds in autumn as a response to short days (SD) and/or decreasing temperatures and flowering in the following spring (Schnablová *et al*., 2021). Such a life cycle is regulated by strong floral repressors that restrict floral transition to certain meristems and time window, conferring seasonal flowering and the maintenance of vegetative meristems for the next yearly cycle (Wang *et al*., 2009, 2011; Albani & Coupland, 2010; Koskela *et al*., 2012; Lazaro *et al*., 2018). Although photoperiod is a major signal controlling seasonal flowering, the underlying mechanisms are poorly understood in perennials (Albani & Coupland, 2010; Andrés & Coupland, 2012).

In perennial woodland strawberry (*Fragaria vesca*), *TERMINAL FLOWER1* (*FvTFL1*) encodes a floral repressor that confers seasonal flowering habit (Koskela *et al*., 2012). The peculiarity of *F. vesca* is that the presence of functional *FvTFL1* reverses the photoperiodic requirement for flower induction. Wild‐type plants require SDs in autumn for flower induction in the temperature range of 13–20°C and flower seasonally, whereas *fvtfl1* mutants are quantitative long‐day (LD) plants that flower perpetually (Heide & Sønsteby, 2007; Koskela *et al*., 2012; Rantanen *et al*., 2015). TFL1 belongs to the family of phosphatidylethanolamine binding proteins (PEBPs) that delay floral transition, promote the indeterminacy of the inflorescence meristem and contribute to the perennial life cycle by blocking the flowering of young axillary shoots (Bradley *et al*., 1996, 1997; Wang *et al*., 2011; Lembinen *et al*., 2023). A gene encoding another PEBP, *FLOWERING LOCUS T* (*FT*), is expressed under flower‐inductive conditions in both LD and SD plants, and FT protein functions as a mobile flower‐inducing signal, the florigen (Kobayashi *et al*., 1999; Corbesier *et al*., 2007; Faure *et al*., 2007; Tamaki *et al*., 2007; Komiya *et al*., 2009; Kong *et al*., 2010). FT is synthesized in leaves and transported to the shoot apical meristem (SAM), where it interacts with the bZIP transcription factor FD to induce flowering (Abe *et al*., 2005). In the SAM, FT has been shown to compete with TFL1 for binding with FD (Zhu *et al*., 2020), providing a possible mechanism for their antagonistic functions. However, in *F. vesca*, expression patterns of *FT*‐homologues suggest a different mechanism in the regulation of photoperiodic flower induction. *FvFT1* is expressed in the leaves specifically in LDs in both wild‐type and *fvtfl1* mutant plants, correlating positively with flowering only in the mutant (Koskela *et al*., 2012; Rantanen *et al*., 2014; Kurokura *et al*., 2017), whereas *FvFT2* and *FvFT3* are highly activated in the late inflorescence and flower meristems (Koskela *et al*., 2017; Lembinen *et al*., 2023).

Homologues of GIGANTEA (GI) and CONSTANS (CO) are major activators of florigens in both SD and LD annual plants (Song *et al*., 2015; Vicentini *et al*., 2023). In Arabidopsis, GI interacts with the blue‐light receptor FLAVIN‐BINDING, KELCH REPEAT, F‐BOX 1 (FKF1) to promote *CO* transcription in LDs (Imaizumi *et al*., 2003; Sawa *et al*., 2007). GI and FKF1 also contribute to the stabilization of CO protein that accumulates when *CO* mRNA expression coincides with light in the afternoon, enabling CO to activate *FT* in leaves (Valverde *et al*., 2004; Hwang *et al*., 2019). A recent work showed that the *CO‐FT/TWIN SISTER OF FT‐LIKE (TSFL)* module promotes flowering in LDs also in the *perpetual flowering1* (*pep1*) mutant of alpine rockcress (*Arabis alpina* L.), a perennial relative of Arabidopsis. Both *AaCO* and *AaFT/TSFL* genes are involved in the regulation of flowering in the primary shoot, whereas *AaCO* is not needed for flowering in axillary shoots, indicating that the photoperiodic pathway contributes also to perennial shoot architecture (Sashidhar & Coupland, 2026). Similarly, *FvCO* and *FvFT1* were shown to promote flowering in LDs in *F. vesca fvtfl1* mutant, and *FvCO* was found to be essential for the activation of *FvFT1* (Koskela *et al*., 2012; Rantanen *et al*., 2014; Kurokura *et al*., 2017). Because these studies in *A. alpina* and *F. vesca* were carried out in perpetual flowering mutants in which LDs promote flowering, they do not provide answers to a major open question: How does photoperiod regulate floral transition in perennials in autumn?

Using transgenic lines and their crosses, we show that LDs activate *FvFT1* through the conserved *FvGI–FvCO–FvFT1* photoperiodic flowering pathway in both seasonal flowering SD genotypes and perpetual flowering LD genotypes of *F. vesca*. We found that in seasonal flowering accessions, LD‐activated FvFT1 upregulates *FvTFL1* to repress flowering, in contrast to its previously reported function as a floral activator in perpetual flowering *fvtfl1* mutant (Koskela *et al*., 2012). Further analysis on European *F. vesca* accessions, that are divided into eastern and western genetic clusters (Toivainen *et al*., 2026), revealed lower average *FvFT1* expression level and earlier flowering in eastern *F. vesca* populations compared with western accessions. Also, a strong positive correlation between *FvFT1* and *FvTFL1* mRNA levels was found, indicating that *FvFT1* is a major driver of geographical differences in flowering time. Overall, our findings define a photoperiodic flowering pathway in strawberries that differs from those described in annual and perennial plants and offers potential strategies to optimize flowering and yield under changing climate in strawberries and related fruit crops of the Rosaceae family.

## Materials and Methods

### Plant material

Woodland strawberry (*Fragaria vesca* L.) accessions Hawaii‐4 (H4, *fvtfl1* mutant), FIN56 (PI551792, originally obtained from the National Clonal Germplasm Repository, Corvallis) and NOR8 from Alta, Norway (70,03244°N, 23,40124°E) were maintained as seeds through self‐pollinations and used for gene functional analyses. FIN56 and NOR8 were previously fully sequenced and showed low genome‐wide heterozygosity levels (Toivainen *et al*., 2026). Newly generated H4‐*FvGI*‐RNAi and H4‐*FvGI* overexpression (OE) lines, and previously reported H4‐*FvFT1*‐RNAi and H4‐*FvCO*‐RNAi lines were germinated from seeds and used for gene functional studies (Koskela *et al*., 2012; Kurokura *et al*., 2017). *FvGI*‐OE lines were generated also in seasonal flowering FIN56 and NOR8 backgrounds. These materials were vegetatively propagated for experiments through runners. To investigate genetic interactions among *FvGI*, *FvCO*, *FvFT1* and *FvTFL1*, several crosses between the transgenic lines and selected accessions were carried out and resulting F_1_ and F_2_ hybrids were used for the experiments (Supporting Information Table S1). Finally, a collection of 181 European *F. vesca* accessions, maintained as clones, was used for studying natural variation in flowering time (Table S2).

### Plasmid constructs and transformation

Plasmid constructs for overexpression lines were created according to Gateway technology with Clonase II (Invitrogen). For *FvGI‐OE* and *FvGI*‐RNAi lines, cDNA sequences from *F. vesca* accession H4 were amplified using primers described in Table S3 and introduced into the pH7WG2D‐1 (overexpression) and pK7GWIWG2‐7F2 (RNAi‐silencing) destination vectors containing green fluorescent protein (GFP) as a positive selection marker (Karimi *et al*., 2002). Strawberry transformation was carried out as described previously (Oosumi *et al*., 2006). Thirteen to twenty transgenic lines were generated and screened for flowering time phenotypes and changes in gene expression for each construct. Three to four lines per construct were selected for further experiments.

### Growth conditions and phenotyping

H4 and its transgenic lines were germinated from seeds on wet filter paper in 18‐h LDs (LD^18h^) at 22°C until cotyledons were visible. At the two‐cotyledon stage, seedlings were rooted to Jiffy Peat Disks (Jiffy Products International BV) in a glasshouse in LD^18h^ at 20°C : 17°C (day : night). After 2 wk, plants with 2–3 leaves were transplanted into 8 × 8 cm pots for photoperiodic treatments. Photoperiodic treatments were conducted in a glasshouse under LD^18h^ (18 h : 6 h, light : dark) or SD^12h^ (12 h : 12 h, light : dark) conditions. In the glasshouse, natural lighting was complemented with 150 μmol m^−2^ s^−1^ photosynthetic photon flux density (PPFD) from high‐pressure sodium lamps (Airam 400W, Kerava, Finland).

Runner plants of NOR8, FIN56 and their transgenic lines were rooted on Jiffy peat disks (Jiffy Products International BV) and then transferred into 8 cm x 8 cm pots filled with peat (Kekkilä, Finland). The plants with at least two to three leaves were subjected to photoperiodic treatments indicated in the figure legends in either glasshouse (FIN56) or growth rooms (NOR8) at 17°C. Growth rooms were equipped with LED lamps (AP67, Valoya, Finland), providing a PPFD of *c*. 150 μmol m^−2^ s^−1^. After the treatments, plants were transferred to the glasshouse for flowering time observations using the conditions described above.

All hybrids were propagated in the glasshouse (LD^18h^ at 20°C : 17°C), and plants were subjected to photoperiodic treatments in either growth rooms described above or in an Arabidopsis chamber AR‐36L2 (Percival, Germany), followed by flowering observations in the glasshouse (LD^18h^ at 20°C : 17°C). Conditions during the photoperiodic treatments are indicated in figure legends.

To explore flowering time of natural accessions, the plants were subjected to SD^12h^ at 16°C for 6 wk in the glasshouse. In another experiment, the accessions were exposed to ambient photoperiod and temperature conditions in Helsinki, Finland, from mid‐August until October 12, 2017 (Field Aug–Oct). Both groups were transferred to the glasshouse (LD^18h^, 20°C : 17°C) after treatments for flowering observations.

All flowering‐time phenotypes were determined as the number of leaves initiated before the first inflorescence and the number of days from the beginning/end of the photoperiodic treatments (indicated in figure legends) to the first flower open.

### RNA extraction and gene expression analyses

Leaf and shoot apex samples were collected under photoperiodic treatments in time points indicated in the figures or figure legends and stored at −80°C before carrying out RNA extraction. Each biological replicate consisted of a single leaflet or one or three shoot apices. Total RNA was extracted from leaf and apex samples as in Mouhu *et al*. (2009). Protoscript II reverse transcriptase kit (NEB, US) was used for cDNA synthesis. Quantitative reverse transcription was performed using a LightCycler 480 SYBR Green I Master kit (Roche Diagnostics, Indianapolis, IN, USA) in a Roche LightCycler 480 (Roche Diagnostics), as described in Mouhu *et al*. (2009). Primers used in this study are shown in Table S3. Each gene was tested by three technical replicates. *FvMSI1* (*FvH4_7g08380*) was used as a stable reference gene (Mouhu *et al*., 2009). The number of biological replicates was described in the figure legends.

### RNA sequencing

For bulk RNA‐seq, total RNA was extracted from leaves collected 4 h after dawn (ZT4) from three biological replicates of 12 accessions (six western and six eastern; Dataset S1) reported in Toivainen *et al*. (2026), grown in a glasshouse under LD^18h^ at 18°C. Libraries were prepared using the TruSeq Total RNA kit (Illumina) following rRNA depletion with Pan‐Plant riboPOOL probes (siTOOLs Biotech) and MagSi‐STA 1.0 beads (Magtivio, Nuth, the Netherlands). Sequencing (100 bp, single‐end) was performed on an Illumina NextSeq500. Raw FASTQ reads were quality‐trimmed with Trimmomatic v.0.39 in single‐end mode (SLIDINGWINDOW:5:20, LEADING:5, TRAILING:5, MINLEN:50). Trimmed reads were aligned to the *F. vesca* reference genome v.4.0 using Star v.2.7.10a with the genome indexed using the v.4.0.a2 annotation from the Genome Database for Rosaceae. Only uniquely mapped reads were retained. Gene‐level counts were generated with HTSeq‐count v.0.13.5 in unstranded mode.

Differentially expressed genes analysis was performed using edgeR (Robinson *et al*., 2010). Gene‐level CPM (counts per million) values from 36 RNA‐seq libraries were obtained using normalized library sizes. Only genes with sufficient counts were retained as determined using filterByExpr function. Three samples were removed from the analysis based on multidimensional scaling plot and sample clustering (Fig. S1). DEGs between the eastern and the western genetic clusters were obtained using the following parameters: fold change > 2, FDR < 0.05, *P*‐value < 0.05.

### Scanning electron microscopy

Dissected meristem samples were fixed overnight in FAA buffer (3.7% formaldehyde, 5% acetic acid and 50% ethanol) and then passed through an ethanol series (50%, 60%, 70%, 80%, 90% and 100%) under mild vacuum (*c*. 0.6 atm). Critical point drying was performed using a Leica EM CPD300 (Leica Mikrosystems GmbH, Vienna, Austria). The dried samples were coated with platinum using a Quorum Q150TS coater (Quorum Technologies, Laughton, UK) and examined using a Quanta 250 FEG scanning electron microscope (FEI, Hillsboro, OR, USA) at the Electron Microscopy Unit, Institute of Biotechnology, University of Helsinki.

### Statistical analyses

For flowering time data in H4 and their transgenic lines, two‐way ANOVA followed by Tukey's HSD multiple comparisons test was carried out. For flowering time data on NOR8 and their transgenic lines, F_1_ and F_2_ hybrids, and European *F. vesca* accessions, one‐way ANOVA followed by Tukey's HSD multiple comparisons or *t*‐tests were performed, as indicated in figure legends. Pearson correlation coefficients were calculated between flowering time and relative gene expression, and among the expression levels of flowering time genes. *P*‐values were estimated with Bonferroni correction. All analyses were performed in R v.4.3.0 (R Core Team, 2023).

### Sequence alignment and phylogenetic analysis

Protein sequences were retrieved from the NCBI database (Table S4). Multiple sequence alignment was performed in MEGA11 using the ClustalW algorithm with default gap opening and extension penalties. The alignment was inspected manually to confirm correct positional homology and adjusted where necessary. Phylogenetic analysis was conducted in MEGA11 using the neighbour‐joining method. Branch support was assessed with 1000 bootstrap replicates, and gaps/missing data were treated using the pairwise‐deletion option. Sequence conservation among the six species, which GI homologue function has been reported, was evaluated using DNAman v.8.0. Conserved regions were identified using the DNAman conservation analysis module, and percent identity was calculated across the full‐length alignment as well as within key conserved blocks, which were shown in different colors.

## Results

### *FvGI* , *FvCO* and *FvFT1* promote flowering in long days in perpetual flowering *F. vesca tfl1* mutant

We have previously identified key regulators of the photoperiodic pathway in *F. vesca* including *FvCO* (FvH4_6g45860; Kurokura *et al*., 2017) and *FvFT1* (FvH4_6g00090; Koskela *et al*., 2012). Here, we analysed a single gene encoding a GI‐like protein from the *F. vesca* genome v.4.0 (FvH4_2g13030). FvGI is highly conserved with GI homologues from other species and is positioned among the GI homologues from other Rosaceous species in the phylogenetic tree (Fig. S2). To explore the role of *FvGI* in the photoperiodic flowering pathway of *F. vesca*, we generated transgenic *FvGI* overexpression (*FvGI*‐OE) and RNAi silencing lines in the perpetual flowering *fvtfl1* mutant accession H4 and examined their flowering phenotypes under LD^18h^ and SD^12h^ conditions together with previously developed *FvCO*‐RNAi and *FvFT1*‐RNAi silencing lines (Koskela *et al*., 2012, Kurokura *et al*., 2017, Fig. S3A–D). Similar to *FvCO*‐RNAi and *FvFT1*‐RNAi plants, *FvGI*‐RNAi lines exhibited a strong late flowering phenotype under LD^18h^ conditions, demonstrating that all these genes are activators of flowering in LDs in H4 (Figs 1a, S4). However, under SD^12h^ conditions, independent *FvGI‐*, *FvCO‐* and *FvFT1‐*RNAi lines flowered either at the same time or slightly later than H4 (Figs 1a, S4). By contrast, *FvGI*‐OE advanced flowering in SD^12h^ but not in LD^18h^ (Figs 1a, S4).

**Fig. 1:**
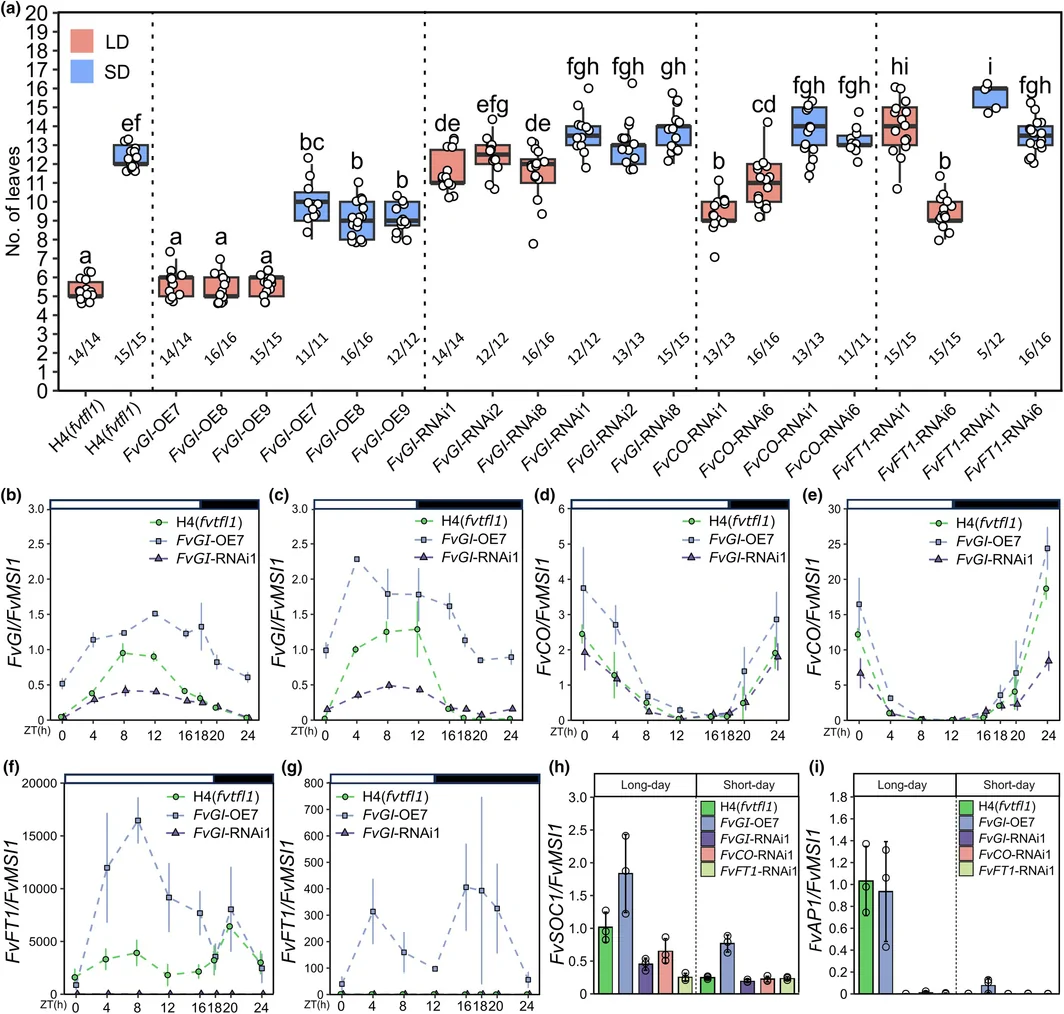
*FvGI*, *FvCO* and *FvFT1* promote flowering in perpetual flowering *Fragaria vesca*. (a) Number of leaves before terminal inflorescence in H4 (*fvtfl1* mutant) and transgenic lines in H4 background under 18‐h long‐day (LD^18h^) and 12‐h short‐day (SD^12h^) conditions. The upper, middle and lower horizontal lines represent the 75^th^ percentile, median and 25^th^ percentile, respectively. The whiskers represent the minimum and maximum. Each point represents the data from individual plants. Numbers under the boxplot indicate the number of flowered plants per the total number of plants in each group. Different letters indicate statistically significant differences as determined by two‐way ANOVA followed by Tukey's HSD multiple comparisons test (*P* < 0.05). (b–g) Relative expression (mean ± SD, *n* = 3) of *FvGI*, *FvCO* and *FvFT1* over a 24‐h cycle in H4 and *FvGI* overexpression (*FvGI*‐OE) and RNAi lines after 3 wk under LD^18h^ (b, d, f) or SD^12h^ (c, e, g) conditions. White and black bars above the panels represent light and dark periods, respectively. (h, i) Relative expression (mean ± SD, *n* = 3) of *FvSOC1* (h) and *FvAP1* (i) in shoot apices of H4, *FvGI‐*OE and *FvGI*, *FvCO* and *FvFT1* RNAi silencing lines. Each biological replicate contains a single shoot apex harvested at ZT3‐ZT5 after 3 wk under LD^18h^ or SD^12h^ treatment.

Consistent with flowering phenotypes, *FvFT1* expression level was very low in the leaves of *FvGI*, *FvCO* or *FvFT1* RNAi lines sampled 4 h after dawn (ZT4) in LD^18h^ (Fig. S3). In the *FvGI*‐OE lines, *FvCO* and *FvFT1* mRNA levels varied, showing either slight to moderate increases or no difference at ZT4 compared with H4. We next analysed the diurnal expression patterns of *FvGI*, *FvCO* and *FvFT1* in H4 and selected transgenic lines. Under LD^18h^ conditions, *FvGI* expression peaked at ZT8‐12, followed by a gradual decline towards the end of the diurnal cycle, while a higher and earlier expression peak (ZT4) was found under SD^12h^ treatment, with a rapid decline towards early night (Fig. 1b,c). In *FvGI*‐OE and RNAi lines, *FvGI* expression remained rhythmic with higher and lower amplitude compared with H4, respectively, under both conditions (Fig. 1b,c). Furthermore, RNAi silencing of *FvCO* or *FvFT1* did not affect *FvGI* expression compared to H4, indicating that they function downstream of FvGI (Fig. S5A,B). *FvCO* expression peaked at dawn/early morning under both conditions in all lines, with higher peaks under SD^12h^ treatment. Its expression was elevated in *FvGI*‐OE lines, while *FvGI*‐RNAi lines showed reduced *FvCO* expression levels at some time points, especially in SD^12h^ (Figs 1d,e, S5C,D). In consistence with the observed flowering phenotypes, *FvFT1* mRNA was detected only under LD^18h^ conditions with a morning peak at ZT4–ZT8 and a higher evening peak at ZT20 in H4, whereas in *FvGI*‐, *FvCO‐* and *FvFT1‐RNAi* lines, *FvFT1* expression remained nearly undetectable under both photoperiods (Figs 1f,g, S5E,F). *FvGI*‐OE plants, by contrast, exhibited highly elevated daytime *FvFT1* mRNA levels under LD^18h^ conditions, while the evening expression peak at ZT20 was not strongly affected. Furthermore, rhythmic *FvFT1* expression with morning and evening peaks was detected also under SD^12h^ conditions, but the overall expression level remained lower than in H4 under LD^18h^ conditions. The expression of *FvFT2* (FvH4_4g30710), that was previously proposed to function as an SD‐activated florigen in *F. vesca* (Gaston *et al*., 2021), was undetectable in all samples (Fig. S5G).

Next, we tested the expression of *FvSOC1* (FvH4_7g12700) and the floral identity gene *FvAP1* (FvH4_4g29600) that were previously shown to be regulated by FvFT1 in the shoot apex (Mouhu *et al*., 2013). Under LD^18h^ conditions, *FvSOC1* mRNA level was slightly elevated in the *FvGI*‐OE7 line and reduced in the *FvGI*, *FvCO* and *FvFT1*‐RNAi lines compared to H4 (Fig. 1h). Consistent with late flowering, *FvAP1* expression was also reduced in all RNAi lines (Fig. 1i). Under SD^12h^ conditions, both *FvSOC1* and *FvAP1* were activated in the early flowering *FvGI*‐OE7 line, while low expression levels were found in H4 and RNAi lines. Taken together, our data suggest that FvGI activates the expression of *FvCO* and *FvFT1* in the leaves, resulting in early floral transition in LDs in H4.

### *FvGI* represses flowering in seasonal flowering *F. vesca*

In contrast to the perpetual flowering H4, seasonal flowering *F. vesca* contains a functional FvTFL1 and requires SDs to induce flowering (FvH4_6g18480; Koskela *et al*., 2012). We next generated *FvGI*‐OE lines in seasonal flowering accessions, including the reference accession FIN56 and the accession NOR8 that exhibited low *FvFT1* expression and early flowering after SD induction (Figs S6A–C, S7A,B). All plants remained vegetative under noninductive LD^18h^ conditions. After flowering‐inductive SD^12h^ treatment, all NOR8 and FIN56 wild‐type plants flowered, whereas most of the *FvGI*‐OE plants remained vegetative (Fig. S7C–F), indicating that FvGI represses flowering in seasonal flowering *F. vesca*. Gene expression analyses confirmed that *FvGI*‐OE strongly activated *FvFT1* expression, both in the morning and in the evening in all lines compared with respective wild‐type lines, while the effect on *FvCO* mRNA level was milder (Fig. S8A–G). In the shoot apex, *FvGI*‐OE lines exhibited higher *FvSOC1* and *FvTFL1* expression levels compared to NOR8 (Fig. S8H–J), indicating that FvGI may repress flowering by activating *FvTFL1* through *FvSOC1* (Mouhu *et al*., 2013).

To examine the repressor function of *FvGI* in more detail, we subjected wild‐type NOR8 and two late flowering *FvGI*‐OE lines to three photoperiods including LD^16h^, SD^12h^ and SD^8h^ for 5 wk, followed by flowering observations in LD^18h^. None of the plants flowered after LD^16h^ (Fig. 2a,b). In SDs, overexpression of *FvGI* shortened the critical photoperiod for flower induction: The SD^8h^ resulted in flowering of 100, 80 and 60% of the NOR8, *FvGI‐*OE9 and *FvGI*‐OE11 plants, respectively, while after SD^12h^, all NOR8 plants flowered, *FvGI*‐OE9 remained vegetative and 20% of the *FvGI*‐OE11 plants flowered. *FvGI*‐OE plants flowered *c*. 1 wk later and with two more leaves than NOR8 after the SD^8h^ treatment. Slower floral transition in *FvGI*‐OE plants was also visible in the meristems. The SEM images, taken at the end of the treatments, showed more developed primary flowers in NOR8 compared with transgenic lines (Fig. S9).

**Fig. 2:**
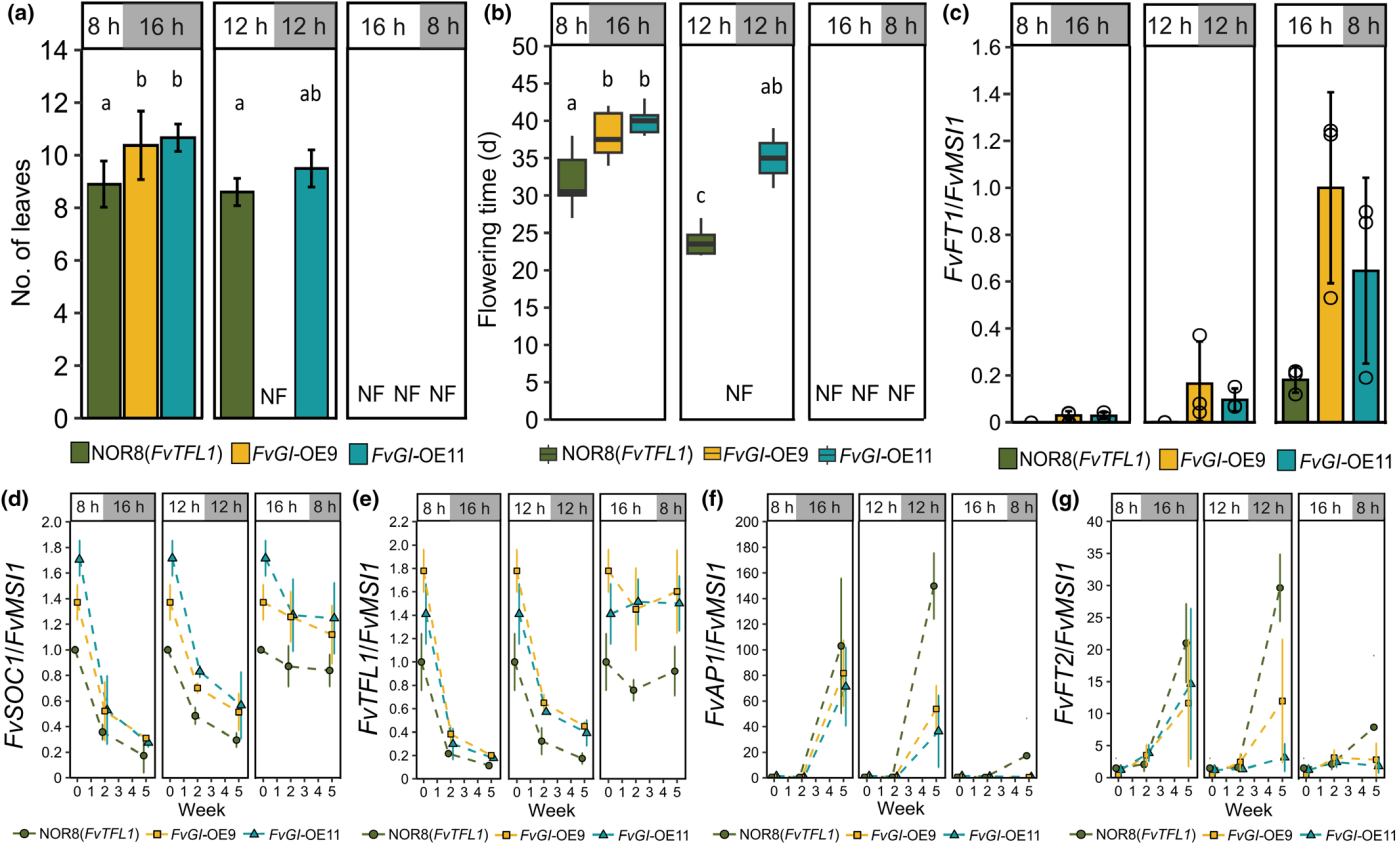
*FvGI* represses flowering in seasonal flowering *Fragaria vesca*. (a) Number of leaves before terminal inflorescence in NOR8 with functional *FvTFL1* alleles and *FvGI* overexpression (*FvGI‐*OE) lines in NOR8 background under indicated conditions. Values indicate mean ± SD (*n* = 10); NF, no flower. (b) Flowering time of NOR8 and *FvGI*‐OE lines indicated as days to anthesis from the end of indicated treatments. Horizontal lines show the 75^th^ percentile (upper), median (middle) and 25^th^ percentile (lower). The whiskers extend to minimum and maximum values. Different letters indicate statistically significant differences as determined by one‐way ANOVA followed by Tukey's HSD multiple comparisons test (*P* < 0.05). Clonally propagated plants were precultured under 18‐h long‐day (LD^18h^) conditions for 4 wk, after which the plants were subjected to 8‐h or 12‐h short days (SD^8h^, SD^12h^) or LD^16h^ at 17°C for 5 wk followed by phenotyping under LD^18h^. (c) *FvFT1* expression (mean ± SD, *n* = 3–4) in leaf samples collected at zeitgeber time 4 (ZT4) after 5 wk under the indicated photoperiods. (d–g) *FvSOC1* (d), *FvTFL1* (e), *FvAP1* (f) and *FvFT2* (g) expression (mean ± SD, *n* = 3) in shoot apices collected from ZT3‐ZT5 after 0, 2 and 5 wk under indicated photoperiods. Three shoot apices were harvested for each biological replicate.

Gene expression analysis showed that *FvFT1* expression increased in leaves of NOR8 and *FvGI*‐OE lines towards longer daylengths, being higher in *FvGI*‐OE lines than in the wild‐type in all photoperiods at ZT4 (Fig. 2c). Higher *FvFT1* expression was associated with elevated *FvSOC1* and *FvTFL1* expression in the shoot apices (Fig. 2d,e). Both *FvSOC1* and *FvTFL1* were downregulated under shorter photoperiods, with the SD^8h^ causing stronger repression than the SD^12h^. The strong downregulation of *FvSOC1* and *FvTFL1* was observed also in *FvGI*‐OE lines under SDs, but their expression levels remained always higher than in the wild‐type. Consistent with flowering results, the floral meristem identity gene *FvAP1* was strongly upregulated in the end of the treatments in all genotypes under SD^8h^ and in NOR8 under SD^12h^ (Fig. 2f). The expression pattern of *FvFT2* closely followed the expression of *FvAP1* in the shoot apex, but its expression was not detected in leaves of NOR8 or its *FvGI*‐OE lines (Figs 2g, S10A,B), suggesting that *FvFT2* may function in early floral development. Taken together, our results indicate that, in seasonal flowering *F. vesca*, *FvGI* activates the expression of *FvFT1* in the leaves and *FvSOC1* and *FvTFL1* in the shoot apex, repressing floral transition in LDs.

### Genetic interactions in the regulation of photoperiodic flowering

Gene expression analyses in transgenic lines suggested that FvGI functions upstream of *FvCO* and *FvFT1* (Figs 1d–g, 2c, S8D–G). To test the genetic interactions of these genes, we crossed NOR8 *FvGI*‐OE line#9 and wild‐type NOR8 (control) with H4, and *FvCO‐* and *FvFT1*‐RNAi lines in H4 background. Half of the NOR8 *FvGI*‐OE × H4 F_1_ hybrids remained vegetative after the SD^12h^ treatment, and the rest of the plants flowered later than NOR8 × H4 controls (Figs 3a,b, S11), confirming that *FvGI*‐OE represses flowering in the hybrids. However, the silencing of *FvCO* or *FvFT1* resulted in equal early flowering in both NOR8 and NOR8 *FvGI*‐OE hybrids, demonstrating that both *FvCO* and *FvFT1* are required for the late flowering of NOR8 *FvGI*‐OE plants. While NOR8 *FvGI*‐OE × H4 hybrids showed elevated *FvFT1* expression levels compared with the NOR8 × H4 hybrids, silencing of *FvCO* rendered *FvFT1* undetectable regardless of *FvGI*‐OE, indicating that the activation of *FvFT1* by FvGI requires *FvCO* (Fig. 3c,d). Furthermore, crossing of NOR8 or NOR8 *FvGI*‐OE line with H4 *FvFT1*‐RNAi line did not affect *FvCO* expression. These results support the presence of *FvGI*‐*FvCO*‐*FvFT1* photoperiodic flowering pathway in *F. vesca*, where *FvCO* and *FvFT1* are fully epistatic to *FvGI*.

**Fig. 3:**
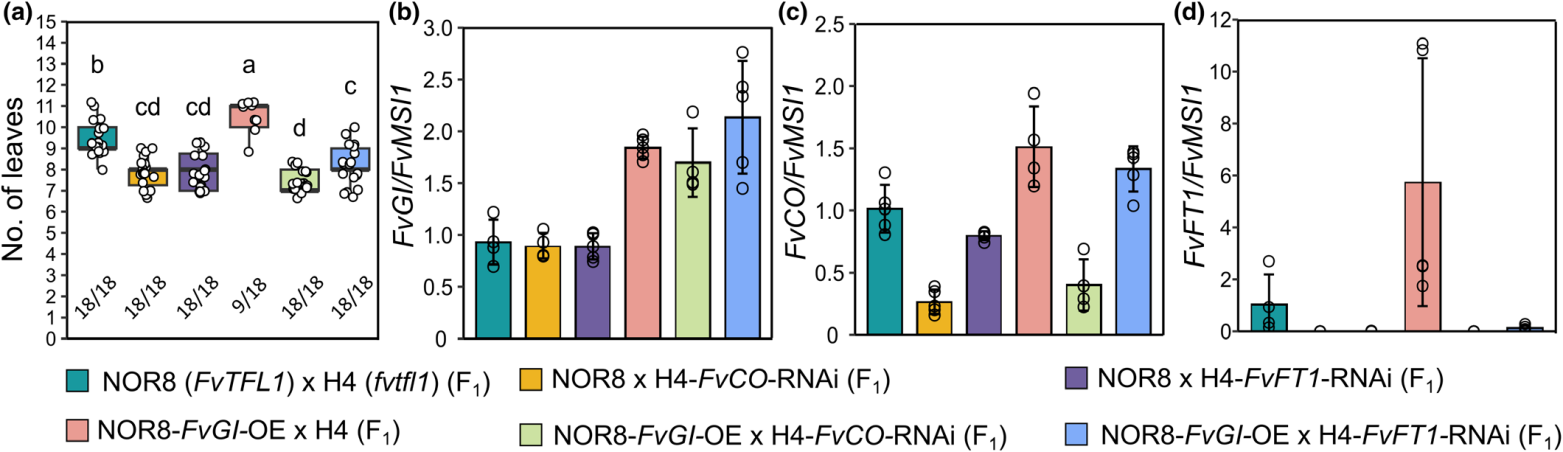
The *FvGI–FvCO–FvFT1* pathway regulates flowering time in seasonal flowering *Fragaria vesca*. (a) Flowering time of F_1_ hybrids of NOR8, H4 and their indicated transgenic lines shown as the number of leaves before the terminal inflorescence. All hybrid plants contain one functional *FvTFL1* allele. The upper, middle and lower horizontal lines indicate the 75^th^ percentile, median and 25^th^ percentile, respectively. The whiskers indicate minimum and maximum values. Each point represents measurement from an individual plant. Four‐week‐old plants, clonally propagated from hybrid seedlings, were subjected to 12‐h short days (SD^12h^) at 17°C for 8 wk followed by phenotyping under 18‐h long days (LD^18h^). OE, overexpression. Numbers under the boxplot indicate the number of flowered plants per the total number of plants in each group. Different letters indicate statistically significant differences as determined by one‐way ANOVA followed by Tukey's HSD multiple comparisons test (*P* < 0.05). (b–d) Relative expression of *FvGI* (b), *FvCO* (c) and *FvFT1* (d) in leaves of F_1_ lines. Leaf samples containing one leaflet per biological replicate were harvested at zeitgeber time 4 under LD^18h^ conditions. Data are mean ± SD (*n* = 4–5).

### *FvTFL1* reverses the effect of *FvFT1* on flowering time

Because *FvGI*‐OE was found to activate *FvFT1* in the leaves and *FvTFL1* in the shoot apex of seasonal flowering *F. vesca* (Fig. 2c,e), we tested if FvFT1 activated *FvTFL1*. To this end, we crossed seasonal flowering accessions, the reference accession FIN56 and the Italian accession IT17 with naturally high *FvFT1* expression level (Fig. S6B), with H4 and H4 *FvFT1*‐RNAi line and analysed the F_1_ hybrids. *FvFT1*‐RNAi hybrids with strongly reduced *FvFT1* mRNA levels flowered early after SD^12h^ treatment, and 40% of IT17 × H4 *FvFT1*‐RNAi and 67% of FIN56 × H4 *FvFT1*‐RNAi plants flowered also under noninductive LD^18h^ conditions (Fig. 4a,b). The expression levels of *FvSOC1* and *FvTFL1* were lower in the shoot apices of *FvFT1*‐RNAi hybrids than in the control plants in LD^18h^ conditions (Figs 4c, S12A,B). After 3 wk of SD^12h^ treatment, *FvTFL1* expression decreased in all plants but remained higher in crosses with the wild‐type H4 compared with H4 *FvFT1*‐RNAi hybrids. At the same time, *FvAP1* exhibited high expression levels only in the *FvFT1*‐RNAi hybrids, confirming that flower induction had happened before this time point in these plants but not in the wild‐type hybrids (Fig. 4d). Taken together, our results suggest that *FvFT1* activates *FvTFL1* expression in the shoot apex to repress flowering in seasonal flowering *F. vesca*, and plants with higher *FvFT1* and *FvTFL1* mRNA levels require longer inductive treatment to reduce the expression level of *FvTFL1* below a critical threshold for floral induction.

**Fig. 4:**
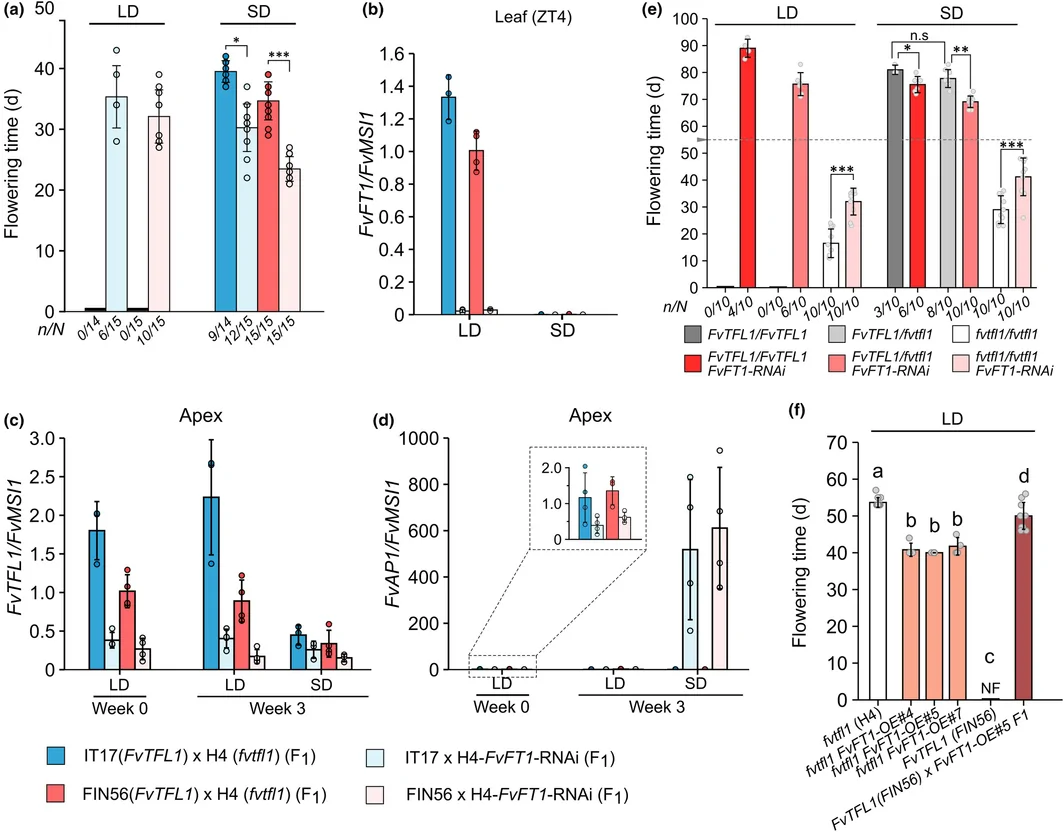
*FvFT1/FvTFL1* epistasis controls flowering in *Fragaria vesca*. (a) Flowering time of F_1_ hybrids between the seasonal flowering genotype IT17 or FIN56 with functional *FvTFL1* alleles and the perpetual flowering genotype H4 (*fvtfl1*) carrying *FvFT1*‐RNAi construct under 18‐h long days (LD^18h^) and after 6 wk under 12‐h short‐day (SD^12h^) conditions. F_1_ crosses with nontransgenic H4 plants were used as controls. Asterisks indicate significant differences between groups at selected time points (***, *P* < 0.001; **, *P* < 0.01; *, *P* < 0.05; *t*‐test). (b–d) Expression of *FvFT1* (b) in leaf samples and *FvTFL1* (c) and *FvAP1* (d) in shoot apices under LD^18h^ conditions and after 3 wk of indicated treatments at zeitgeber time 4 (ZT4) (b) or ZT3‐ZT5 (c, d) time points. Inset panel in (d) shows a close view of initial (before the start of treatment) expression levels of *FvAP1* at week 0. (e) Flowering time of IT17 × *FvFT1*‐RNAi F_2_ plants segregating for *FvFT1‐*RNAi construct and *FvTFL1* and *fvtfl1* alleles after 8 wk under SD^12h^ conditions. *y*‐axis shows days since the beginning of the treatments; dashed grey line marks the end of the indicated treatments after which all the plants were moved to LD^18h^ conditions. Numbers under *x*‐axis denote the number of flowered plants (*n*) and total number of plants (*N*) in each group. (f) Flowering time of H4 and FIN56 wild‐type plants and their *FvFT1* overexpression (*FvFT1*‐OE) lines under LD^18h^ conditions. In (e, f), data are mean ± SD (*n* = 10). Asterisks (e) or different letters (f) indicate the significant differences between selected groups (***, *P* < 0.001; **, *P* < 0.01; *, *P* < 0.05; Tukey's HSD test).

To obtain direct evidence of the interaction of the photoperiodic pathway with *FvTFL1* we segregated *FvTFL1* alleles and *FvFT1‐*RNAi insertion in IT17 × H4 *FvFT1*‐RNAi F_2_ lines and observed the flowering time. Under both LD^18h^ and SD^12h^ conditions, *fvtfl1* homozygous mutants with the *FvFT1*‐RNAi construct flowered later than the *fvtfl1* mutants without the *FvFT1‐*RNAi construct, confirming that FvFT1 activates flowering in the absence of functional FvTFL1 (Fig. 4e). However, the presence of one or two alleles encoding functional FvTFL1 resulted in SD‐dependent flowering and reverted the function of FvFT1; the plants with the *FvFT1*‐RNAi construct flowered earlier than the controls. In addition, approximately half of the *FvFT1*‐silenced plants homozygous or heterozygous for functional FvTFL1 flowered even under noninductive LD conditions. A higher proportion of flowering plants and earlier flowering of *FvTFL1/fvtfl1* heterozygotes compared with plants containing two wild‐type alleles supported the dosage‐dependent effect of *FvTFL1* on flowering in *F. vesca* (Fig. 4e).

Altogether, our results indicate that flowering regulation in *F. vesca* involves an epistatic interaction between the *FvGI‐FvCO‐FvFT1* photoperiodic pathway and *FvTFL1*. When the functional *FvTFL1* allele is present, FvFT1 prevents flowering in LDs by promoting *FvTFL1* expression, while in the absence of functional *FvTFL1*, FvFT1 functions as a florigen that activates the expression of *FvAP1*, thus promoting flowering. Even in plants with functional FvTFL1, FvFT1 retains florigenic activity, as its RNAi silencing slightly reduces *FvAP1* expression (Figs 4d, S12C), but this activity is masked by the *FvTFL1* activation. Moreover, the expression of *FvFT1* specifically in the leaves (Koskela *et al*., 2012) is critical for the flowering repression mechanism presented here because ectopic expression of *FvFT1* by the 35S promoter induces flowering even in the presence of *FvTFL1* (Fig. 4f).

### *FvFT1* contributes to natural variation in flowering time

Finally, we explored natural flowering time variation in a European collection of *F. vesca* accessions that were recently shown to form separate eastern and western genetic clusters occupying different climatic niches (Toivainen *et al*., 2026). After flower induction under SD^12h^ in glasshouse or field conditions in autumn in Helsinki, a wide variation in flowering time was observed, with the eastern accessions flowering significantly earlier than the western ones, suggesting adaptive variation (Fig. 5a–c).

**Fig. 5:**
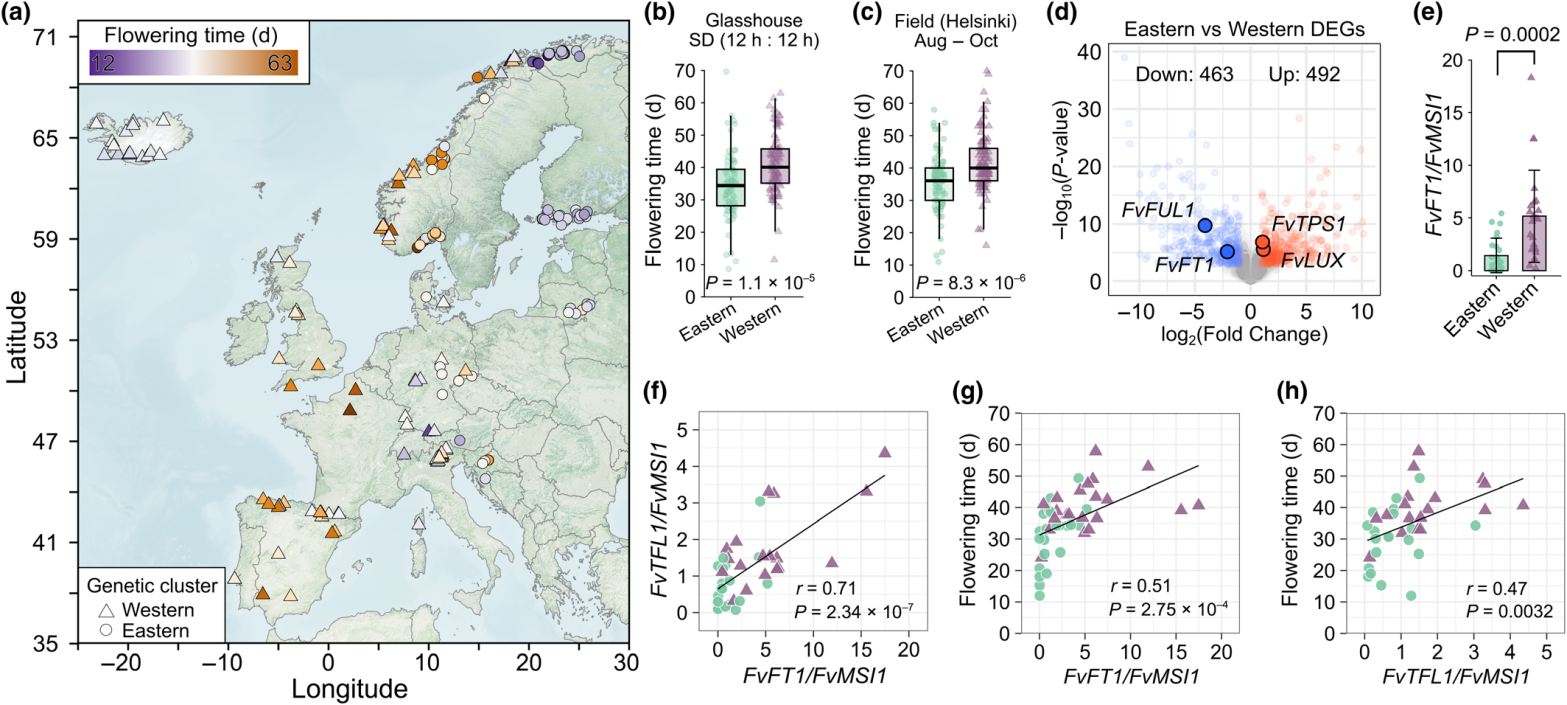
Natural variation of flowering time correlates with *FvFT1* and *FvTFL1* expression in European *Fragaria vesca* accessions. (a) Map showing collection sites of woodland strawberry accessions and their flowering time and genetic clusters. Accessions were divided into eastern and western genetic clusters according to Toivainen *et al*. (2026). (b, c) Boxplots showing differences in flowering time between the accessions of eastern and western genetic clusters after flower induction under 12‐h short‐day (SD^12h^) conditions in a glasshouse (b; 179 accessions) or in the field (c; 181 accessions). The upper, middle and lower horizontal lines represent the 75^th^ percentile, median and 25^th^ percentile, respectively. The whiskers represent the minimum and maximum. Each point represents the mean value for the individual accession (*n* = 2–5). (d) Differentially expressed genes (DEGs) in leaves of six western and six eastern accessions collected under 18‐h long days (LD^18h^) at zeitgeber time 4 (ZT4). (e) Mean expression levels of *FvFT1* in 25 eastern and 23 western accessions (dots) (LD^18h^, ZT4). Error bars represent ±SD. *P*‐values in (b, c, e) were estimated using Student's *t*‐test. (f) Pearson's correlation between the relative expression of *FvFT1* (data from panel e) in leaves and *FvTFL1* in shoot apices (LD^18h^, ZT3‐5) in 21 eastern and 19 western accessions. (g, h) Pearson's correlation between flowering time and *FvFT1* (g) or *FvTFL1* (h) expression levels using the expression data shown in (e, f). *P*‐values in (f–h) were estimated using Pearson's correlation test, Bonferroni adjusted.

RNA sequencing from leaves of six eastern and six western accessions grown under LD^18h^ conditions identified 955 genes differentially expressed between the genetic clusters (Fig. S1; Dataset S1). Among these, four candidate flowering time genes were found including *FvTPS1* (*TREHALOSE‐6‐PHOSPHATE SYNTHASE1*, FvH4_3g06590), *FvLUX* (*LUX ARRHYTHMO*, FvH4_3g36890), *FvFUL1* (*FRUITFUL*, FvH4_5g13500) and *FvFT1* (Figs 5d, S13A). Both *LUX* and *TPS1* are known to affect *FT* expression in Arabidopsis (Hazen *et al*., 2005; Wahl *et al*., 2013) and an FUL homolog in barley (Sasani *et al*., 2009), while their functions in *F. vesca* remain to be clarified. The expression of *FvFT1* was typically lower in the accessions of the earlier flowering eastern cluster (Fig. S13A). We verified this by RT‐qPCR analysis in a larger set of accessions (25 eastern and 23 western), spanning the geographical range of European *F. vesca*, and found higher *FvFT1* expression levels in the western than the eastern accessions at ZT4 under LD^18h^ conditions (Fig. 5e). Furthermore, we found a strong correlation in *FvFT1* expression levels between ZT4 and ZT16 time points (Fig. S6C), suggesting that the observed differences at ZT4 are not time point specific. The analysis of shoot apex samples revealed that the expression of *FvFT1* in leaves of accessions correlated with *FvTFL1* expression levels in the shoot apex under LD^18h^ conditions (Fig. 5f). Increasing expression of both genes under LD^18h^, before flower inductive conditions, was associated with delayed flowering after the subsequent flower induction under SD^12h^ conditions or in the field in autumn (Figs 5g,h, S13B). Although *FvGI* and *FvCO* expression levels did not consistently differ between the two clusters in the RNAseq data (Fig. S13A), their expressions were correlated among a larger set of accessions, as were *FvCO* and *FvFT1* (Fig. S13C). Altogether, these findings indicate that the *FvGI–FvCO–FvFT1–FvTFL1* photoperiodic pathway contributes to flowering time variation across geographic and climatic ranges in *F. vesca* accessions, with strong correlations between flowering time and latitude or winter temperature of plant collection sites (Fig. S13D).

## Discussion

Here, we studied photoperiodic flowering in *F. vesca*, a perennial species with a wide geographical distribution in the northern hemisphere (Hilmarsson *et al*., 2017; Toivainen *et al*., 2026). Previous studies showed that the floral repressor FvTFL1 confers the SD requirement of flower induction in *F. vesca*, while the *fvtfl1* mutant flowers early in LDs (Koskela *et al*., 2012). By testing *FvGI* function and its genetic interactions with previously reported genes (Koskela *et al*., 2012; Kurokura *et al*., 2017), we show that the *FvGI‐FvCO‐FvFT1* photoperiodic pathway expressed in the leaves is conserved in *F. vesca*. This pathway activates LD‐flowering in the *fvtfl1* mutant, while in seasonal flowering, wild‐type plants the pathway is rewired to activate *FvTFL1* that represses flowering in the shoot apex, reversing the photoperiodic response. Due to this connection, wild‐type plants require SDs for flower induction that occurs after the downregulation of *FvFT1* and *FvTFL1* in autumn. In natural accessions, *FvFT1* expression correlates positively with *FvTFL1* mRNA levels and is associated with differences in flowering time in plants collected across Europe, suggesting that a flower‐repressive photoperiodic pathway may contribute to flowering time adaptation across geographic and climatic ranges.

### Epistatic interaction between *FvFT1* and *FvTFL1* reverses photoperiodic response

Homologues of FT have been shown to induce flowering in both LD and SD plants (Kobayashi *et al*., 1999; Faure *et al*., 2007; Tamaki *et al*., 2007; Komiya *et al*., 2009; Kong *et al*., 2010), while TFL1 represses flowering (Bradley *et al*., 1997; Wang *et al*., 2011), possibly through competing for binding with a bZIP transcription factor FD that is needed for the regulation of their target genes (Zhu *et al*., 2020). Multiple lines of evidence suggest different mechanisms in the regulation of photoperiodic flowering in *F. vesca: FvFT1* is expressed in leaves in LDs when *FvTFL1* is also activated in the shoot apex to repress flowering, and floral transition occurs in SDs when *FvTFL1* is downregulated and *FvFT1* expression is undetectable (Koskela *et al*., 2012). In *fvtfl1* mutant, however, *FvFT1* promotes flowering in LDs and is fully responsible for this photoperiodic response (Koskela *et al*., 2012; Rantanen *et al*., 2014; Kurokura *et al*., 2017). By segregating *FvTFL1* alleles and the *FvFT1*‐RNAi insertion in an F_2_ crossing population we demonstrated that *FvTFL1* is epistatic to *FvFT1* in the photoperiodic flowering. All F_2_ lines homozygous for *fvtfl1* alleles flowered early in LDs, with RNAi‐silencing of *FvFT1* delaying flowering confirming that FvFT1 promotes flowering in the *fvtfl1* mutant. By contrast, when one or two functional *FvTFL1* alleles were present, flowering became SD‐dependent, and the effect of *FvFT1* was reversed to a repressor of flowering. *FvFT1*‐RNAi plants flowered earlier than controls after the SD treatment, and a substantial proportion of *FvFT1*‐RNAi plants flowered even in noninductive LDs.

To understand the mechanism of the epistatic interaction between *FvFT1* and *FvTFL1*, we studied gene expression in *FvFT1*‐RNAi lines and natural accessions of *F. vesca*. We found lower expression level of *FvTFL1* in *FvFT1*‐RNAi lines under both LD^18h^ and SD^12h^ conditions compared with wild‐type plants and a positive correlation between *FvFT1* and *FvTFL1* mRNA levels in natural accessions, suggesting that FvFT1 activates *FvTFL1* in the SAM under LDs to repress flowering in *F. vesca*. Further studies are needed to understand how this activation occurs at the molecular level, including a role of FvSOC1 that was previously found to activate *FvTFL1* expression (Mouhu *et al*., 2013) (Figs 1h, S8F–I). Recent work in Arabidopsis showed that FT‐activated LEAFY (LFY) upregulated *TFL1* to maintain indeterminate inflorescence meristem (Huang *et al*., 2026). Further analysis of four *LFY* genes present in *F. vesca* genome is needed for testing if LFY has a similar role in *F. vesca* (Zhang *et al*., 2023).

Several lines of evidence suggested that the *FvFT1/FvTFL1* module has a quantitative effect on floral transition and flowering time: (1) *FvFT1*‐RNAi lines with low *FvFT1* and *FvTFL1* expression levels showed early upregulation of floral marker gene *FvAP1* under SDs and early flowering compared with wild‐type plants (Fig. 4), (2) *FvGI*‐OE lines with increased *FvFT1* and *FvTFL1* mRNA levels required shorter photoperiod for floral transition and flowered later than wild‐type (Fig. 2), and (3) *FvTFL1/fvtfl1* heterozygote plants flowered earlier than *FvTFL1* homozygotes (Samad *et al*., 2017, Fig. 4e). Overall, these data support the hypothesis that the level of *FvFT1/FvTFL1* expression drives the strength of floral repression in LDs and affects SD requirements of floral transition. However, the mechanism of flower induction in SDs remains elusive. Previous study found the expression of *FvFT2* in leaves and suggested that FvFT2 could induce flowering in SDs (Gaston *et al*., 2021). We could not detect *FvFT2* expression in the leaves of tested accessions and transgenic lines under any conditions, but *FvFT2* was upregulated in the shoot apex in parallel with *FvAP1* indicating that it plays a role in early floral development instead of floral induction. Possible role of SQUAMOSA PROMOTER‐BINDING PROTEIN‐LIKE (SPL) transcription factors that induce flowering in SDs in *A. thaliana* and *A. alpina* (Roggen *et al*., 2025) should be tested in *F. vesca*.

### *FvGI‐FvCO‐FvFT1* pathway controls photoperiodic flowering

*CO/FT* module has been shown to control photoperiodic flowering in annual models Arabidopsis and rice (Valverde *et al*., 2004; Ishikawa *et al*., 2011), and among perennials, in perpetual flowering mutants of *F. vesca* and *A. alpina* (*fvtfl1* and *pep1* mutants, respectively; Kurokura *et al*., 2017; Sashidhar & Coupland, 2026). Here, we extended these studies to *FvGI* and demonstrated that all three genes are required for the photoperiodic flowering of *F. vesca*. In RNAi lines of *FvGI*, *FvCO* and *FvFT1* in H4 background (*fvtfl1* mutant; Koskela *et al*., 2012), *FvFT1* expression levels remained below/close to the detection limit in LDs, and the lines almost completely lost the early LD flowering observed in H4. *FvGI*‐OE, by contrast, increased *FvFT1* expression in leaves resulting in early flowering specifically in SDs in H4 background, and a similar SD‐specific response was previously reported in *FvCO*‐OE lines (Kurokura *et al*., 2017). The effect of increasing *FvFT1* expression seems to be quantitative; SD‐grown H4 with undetectable *FvFT1* expression flowers late, mild activation of *FvFT1* in *FvGI*‐OE plants in SD results in intermediate flowering time, while H4 grown in LDs flowers early because of several times higher *FvFT1* mRNA levels compared with *FvGI*‐OE plants grown in SDs (Fig. 1f,g). The florigenic effect of *FvFT1* is possibly saturated in H4 grown under LD^18h^ conditions because *FvGI*‐OE does not advance flowering further in LDs. However, *FvFT1*‐OE plants with constitutive *FvFT1* expression lose their photoperiodic response and plants flower early under both SD and LD conditions (Fig. 4f; Rantanen *et al*., 2014).

Overexpression of *FvGI* increased *FvFT1* expression also in plants possessing functional *FvTFL1* (NOR8, FIN56), but resulted in delayed SD‐flowering, consistent with the epistatic effect of *FvTFL1* to *FvFT1*. Furthermore, the late flowering phenotype of NOR8 *FvGI*‐OE plants was completely reversed by silencing of *FvCO* or *FvFT1* that also reduced *FvFT1* expression close to the detection limit, demonstrating that *FvCO* and *FvFT1* were epistatic to *FvGI*. Together with previous research on *FvCO* function (Kurokura *et al*., 2017), these findings established the *FvGI‐FvCO‐FvFT1* pathway with a major role in mediating the LD signal to activate flowering in *F. vesca fvtfl1* mutants. In wild‐type plants, this pathway activates *FvTFL1*, and SDs are required to suppress the whole pathway to induce flowering. In other SD plants, different mechanisms repress flowering in LDs. In rice, Ghd7 blocks *Hd1* (CO‐homolog) function and in soybean, E1 and GmCOL1a/b repress *FT2a/5a* and flowering (Xia *et al*., 2012; Cao *et al*., 2015; Xu *et al*., 2015; Zong *et al*., 2021).

Sequences and diurnal expression rhythms of *GI* homologs are largely conserved in both LD and SD plants, suggesting broadly conserved roles in photoperiodic signalling (Fowler *et al*., 1999; Hayama *et al*., 2003; Hecht *et al*., 2007; Li *et al*., 2013; Deng *et al*., 2015, Fig. S2). In Arabidopsis, GI promotes *CO* transcription in LDs, allowing CO protein to peak in the evening to activate *FT* (Valverde *et al*., 2004; Sawa *et al*., 2007). However, *FvCO* mRNA peaks at dawn with a broader expression phase in LDs, the pattern resembling that of Arabidopsis *COL1/COL2*, pea *PsCOLa* and soybean *GmCOL1a/b* (Ledger *et al*., 2001; Hecht *et al*., 2007; Wu *et al*., 2014). This *FvCO* expression pattern is needed for normal *FvFT1* expression peaks in the morning and late evening, because both peaks are lost in *FvCO* RNAi lines (Kurokura *et al*., 2017). Similarly, *FvFT1* expression peaks were lost in *FvGI*‐RNAi lines, although *FvCO* mRNA levels were not clearly affected, indicating that FvGI regulates *FvFT1* expression mainly through posttranscriptional regulation of FvCO, which is further supported by the observations that both *FvGI*‐OE and *FvCO*‐OE had only a weak effect on *FvFT1* mRNA levels in SDs, compared with LDs (Kurokura *et al*., 2017, Fig. 1b–g). Such a mechanism is consistent with an LD‐dependent stabilization of CO in Arabidopsis that involves FKF1 and GI (Valverde *et al*., 2004; Song *et al*., 2012, 2014; Hwang *et al*., 2019). In conclusion, we show strong evidence for the *FvGI–FvCO–FvFT1* photoperiodic pathway in *F. vesca*. Further studies are needed to understand posttranscriptional regulation of FvCO and a possible role of *FvFKF1*, that has overlapping expression phase with *FvGI* (Kurokura *et al*., 2017), in photoperiodic flowering of *F. vesca*.

### Flowering time variation in European *F. vesca*

*Fragaria vesca* occupies a wide geographical range from Mediterranean to arctic region in the northern hemisphere (Hilmarsson *et al*., 2017). Our data indicate that flowering time variation in *F. vesca* accessions collected across Europe is associated with their population structure, which consists of two major genetic clusters. These western and eastern clusters originate from different glacial refugia and inhabit distinct climates across a temperature seasonality gradient (Toivainen *et al*., 2026). We found that eastern accessions flowered significantly earlier than western accessions under both SD and field conditions, with flowering time most strongly correlating with mean annual and winter temperatures of plant collection sites. Also, the latitudinal correlation was strong, with accessions from arctic regions, particularly in the east, showing extreme early flowering. Such a pattern of flowering time variation can be attributed to the length of the growing season that creates strong selection pressure on reproductive traits (Austen *et al*., 2017), suggesting adaptive divergence in flowering regulation.

Reduced activity of the photoperiodic pathway was suggested to contribute to adaptation to short growing seasons in both annual and perennial plants (Hyun *et al*., 2019). Consistent with this idea, we found that decreasing expression levels of *FvFT1* and *FvTFL1* under LD^18h^ conditions correlated with earlier flowering after subsequent flower induction treatments in European *F. vesca* accessions. Although the measured LD expression states of *FvFT1* and *FvTFL1* do not mechanistically govern what happens during SD induction, together with transgenic data discussed above, they support the hypothesis that the strength of the *FvFT1/FvTFL1*‐driven LD repression system may contribute to flowering time variation. While *FvFT1* and *FvTFL1* mRNA levels showed a strong positive correlation with clear association with genetic clusters, weaker positive *FvGI/FvCO* and *FvCO/FvFT1* correlations were found, and there was no clear east–west pattern in *FvGI* or *FvCO* expression, suggesting that *FvFT1* expression variation associated with geography and population structure depends on mechanisms other than the regulation of *FvGI* or *FvCO* transcription. However, arctic accessions (NOR14 and NOR15) from the highly differentiated Kåfjord population (Toivainen *et al*., 2026), that flowered first in different experiments, exhibited clearly reduced *FvCO* expression and extremely low *FvFT1* mRNA levels, reflecting population‐specific responses to photoperiods. A previous study showed substantial shifts in the timing of *GI* expression between natural Arabidopsis accessions (De Montaigu *et al*., 2015), highlighting the importance of studying multiple time points when comparing natural accessions. Although we found a strong correlation between ZT4 and ZT16 expression levels of *FvFT1* among 13 accessions, more detailed analysis is needed to explore possible differences in the circadian phase, entrainment, or waveform timing of flowering genes that may contribute to observed flowering time variation. While FvGI and FvCO play major roles as activators of *FvFT1* expression, studies on additional candidate genes, including *FvTPS1*, *FvLUX* and *FvFUL1* identified in our RNA sequencing work, are needed for more detailed understanding of natural flowering time variation and underlying regulatory network in *F. vesca*. Other mechanisms may include *cis*‐regulatory variation of *FT* that has been reported to contribute to flowering time variation in Arabidopsis and soybean (Liu *et al*., 2014; Rosas *et al*., 2014; Bao *et al*., 2019; Chen *et al*., 2020).

In conclusion, *FvGI* and *FvCO* are needed for the activation of *FvFT1* in leaves in LDs, and they constitute a major photoperiodic response pathway of the species. In the perpetual flowering *fvtfl1* mutant, this pathway activates *FvSOC1* and *FvAP1* in the shoot apex, resulting in rapid flowering in LDs (Fig. 1; Kurokura *et al*., 2017). Functional *FvTFL1*, that is present in all natural accessions tested in this work, reverses the photoperiodic response (Koskela *et al*., 2012) because the photoperiodic pathway activates *FvTFL1* at least partially through *FvSOC1*, overriding *FvAP1* activation and repressing flowering (Fig. 4; Mouhu *et al*., 2013). Finally, in natural accessions, expression levels of *FvFT1* and *FvTFL1* were associated with geographic flowering time variation across Europe, with decreasing *FvFT1* and *FvTFL1* mRNA levels and earlier flowering towards north and east. Further studies are needed to explore possible contributions of these genes to flowering time adaptation across climatic gradients.

## Competing interests

None declared.

## Author contributions

QZ, SL, EK and TH designed the research. QZ, SL, TT, TK and GF carried out the experimental work. SL, TT and GF analysed RNA sequencing data. TH, EK and PE supervised the study. The manuscript was written by QZ and TH with input from all authors.

## Disclaimer

The New Phytologist Foundation remains neutral with regard to jurisdictional claims in maps and in any institutional affiliations.

## Supporting information


**Dataset S1** RNA sequencing data of 12 *Fragaria vesca* accessions.

**Fig. S1** Clustering of RNA sequencing samples.
**Fig. S2** Phylogenetic analysis and multiple sequence alignment of GIGANTEA homologs.
**Fig. S3** Gene expression in *Fragaria vesca* accession H4 and transgenic lines.
**Fig. S4** Flowering time of *Fragaria vesca* accession H4 and indicated transgenic lines.
**Fig. S5** Diurnal gene expression rhythms in *Fragaria vesca* accession H4 and transgenic lines.
**Fig. S6** Flowering time and *FvFT1* expression in natural accessions of *Fragaria vesca*.
**Fig. S7** Gene expression and phenotypes of indicated *Fragaria vesca* genotypes.
**Fig. S8** Expression of flowering time genes in *Fragaria vesca* accession NOR8 and *FvGI* overexpression lines.
**Fig. S9** Scanning electron microscope images of *Fragaria vesca* meristems.
**Fig. S10** Expression of *FvFT1* and *FvFT2* in *Fragaria vesca* accession NOR8 and *FvGI* overexpression lines.
**Fig. S11** Flowering time of indicated *Fragaria vesca* hybrids.
**Fig. S12** Expression of flowering genes in indicated *Fragaria vesca* hybrids.
**Fig. S13** Comparison of gene expression levels in *Fragaria vesca* accessions and correlations between gene expression, flowering time and bioclimatic and geographical variables.

**Table S1** *Fragaria vesca* hybrids used in this study.
**Table S2** *Fragaria vesca* accessions used in this study.
**Table S3** Primers used in this study.
**Table S4** Protein sequences used for the phylogenetic analysis of GI.
Please note: Wiley is not responsible for the content or functionality of any Supporting Information supplied by the authors. Any queries (other than missing material) should be directed to the *New Phytologist* Central Office.

## Data Availability

All the raw sequence reads from RNA sequencing are available at NCBI Sequence Read Archive under Bio‐project ID PRJNA1503464.

## References

[nph71555-bib-0001] Abe M , Kobayashi Y , Yamamoto S , Daimon Y , Yamaguchi A , Ikeda Y , Ichinoki H , Notaguchi M , Goto K , Araki T . 2005. FD, a bZIP protein mediating signals from the floral pathway integrator FT at the shoot apex. Science 309: 1052–1056. 10.1126/science.1115983 16099979

[nph71555-bib-0002] Albani MC , Coupland G . 2010. Comparative analysis of flowering in annual and perennial plants. Current Topics in Developmental Biology 91: 323–348. 10.1016/S0070-2153(10)91011-9 20705187

[nph71555-bib-0003] Andrés F , Coupland G . 2012. The genetic basis of flowering responses to seasonal cues. Nature Reviews Genetics 13: 627–639. 10.1038/nrg3291 22898651

[nph71555-bib-0004] Austen EJ , Rowe L , Stinchcombe JR , Forrest JR . 2017. Explaining the apparent paradox of persistent selection for early flowering. New Phytologist 215: 929–934. 10.1111/nph.14580 28418161

[nph71555-bib-0005] Bao S , Hua C , Huang G , Cheng P , Gong X , Shen L , Yu H . 2019. Molecular basis of natural variation in photoperiodic flowering responses. Developmental Cell 50: 90–101. 10.1016/j.devcel.2019.05.018 31178399

[nph71555-bib-0006] Bradley D , Carpenter R , Copsey L , Vincent C , Rothstein S , Coen E . 1996. Control of inflorescence architecture in Antirrhinum. Nature 379: 791–797. 10.1038/379791a0 8587601

[nph71555-bib-0007] Bradley D , Ratcliffe O , Vincent C , Carpenter R , Coen E . 1997. Inflorescence commitment and architecture in Arabidopsis. Science 275: 80–83. 10.1126/science.275.5296.80 8974397

[nph71555-bib-0008] Cao D , Li Y , Lu S , Wang J , Nan H , Li X , Shi D , Fang C , Zhai H , Yuan X . 2015. GmCOL1a and GmCOL1b function as flowering repressors in soybean under long‐day conditions. Plant and Cell Physiology 56: 2409–2422. 10.1093/pcp/pcv152 26508522

[nph71555-bib-0009] Chen L , Cai Y , Qu M , Wang L , Sun H , Jiang B , Wu T , Liu L , Sun S , Wu C . 2020. Soybean adaption to high‐latitude regions is associated with natural variations of GmFT2b, an ortholog of FLOWERING LOCUS T. Plant, Cell & Environment 43: 934–944. 10.1111/pce.13695 PMC7154755 31981430

[nph71555-bib-0010] Corbesier L , Vincent C , Jang S , Fornara F , Fan Q , Searle I , Giakountis A , Farrona S , Gissot L , Turnbull C *et al*. 2007. FT protein movement contributes to long‐distance signaling in floral induction of Arabidopsis. Science 316: 1030–1033. 10.1126/science.1141752 17446353

[nph71555-bib-0011] De Montaigu A , Giakountis A , Rubin M , Tóth R , Cremer F , Sokolova V , Porri A , Reymond M , Weinig C , Coupland G . 2015. Natural diversity in daily rhythms of gene expression contributes to phenotypic variation. Proceedings of the National Academy of Sciences, USA 112: 905–910. 10.1073/pnas.1422242112 PMC4311856 25548158

[nph71555-bib-0012] Deng W , Clausen J , Boden S , Oliver SN , Casao MC , Ford B , Anderssen RS , Trevaskis B . 2015. Dawn and dusk set states of the circadian oscillator in sprouting barley (*Hordeum vulgare*) seedlings. PLoS ONE 10: e0129781. 10.1371/journal.pone.0129781 PMC4465908 26068005

[nph71555-bib-0013] Faure S , Higgins J , Turner A , Laurie DA . 2007. The FLOWERING LOCUS T‐like gene family in barley (*Hordeum vulgare*). Genetics 176: 599–609. 10.1534/genetics.106.069500 PMC1893030 17339225

[nph71555-bib-0014] Fowler S , Lee K , Onouchi H , Samach A , Richardson K , Morris B , Coupland G , Putterill J . 1999. GIGANTEA: a circadian clock‐controlled gene that regulates photoperiodic flowering in Arabidopsis and encodes a protein with several possible membrane‐spanning domains. EMBO Journal 18: 4679–4688. 10.1093/emboj/18.17.4679 PMC1171541 10469647

[nph71555-bib-0015] Gaston A , Potier A , Alonso M , Sabbadini S , Delmas F , Tenreira T , Cochetel N , Labadie M , Prévost P , Folta KM . 2021. The FveFT2 florigen/FveTFL1 antiflorigen balance is critical for the control of seasonal flowering in strawberry while FveFT3 modulates axillary meristem fate and yield. New Phytologist 232: 372–387. 10.1111/nph.17557 PMC8519138 34131919

[nph71555-bib-0016] Hayama R , Yokoi S , Tamaki S , Yano M , Shimamoto K . 2003. Adaptation of photoperiodic control pathways produces short‐day flowering in rice. Nature 422: 719–722. 10.1038/nature01549 12700762

[nph71555-bib-0064] Hazen SP , Schultz TF , Pruneda‐Paz JL , Borevitz JO , Ecker JR , Kay SA . 2005. *LUX ARRHYTHMO* encodes a Myb domain protein essential for circadian rhythms. Proceedings of the National Academy of Sciences, USA 102: 10387–10392. 10.1073/pnas.0503029102 PMC1177380 16006522

[nph71555-bib-0017] Hecht V , Knowles CL , Vander Schoor JK , Liew LC , Jones SE , Lambert MJ , Weller JL . 2007. Pea LATE BLOOMER1 is a GIGANTEA ortholog with roles in photoperiodic flowering, deetiolation, and transcriptional regulation of circadian clock gene homologs. Plant Physiology 144: 648–661. 10.1104/pp.107.096818 PMC1914181 17468223

[nph71555-bib-0018] Heide OM , Sønsteby A . 2007. Interactions of temperature and photoperiod in the control of flowering of latitudinal and altitudinal populations of wild strawberry (*Fragaria vesca*). Physiologia Plantarum 130: 280–289.

[nph71555-bib-0019] Hilmarsson HS , Hytönen T , Isobe S , Göransson M , Toivainen T , Hallsson JH . 2017. Population genetic analysis of a global collection of *Fragaria vesca* using microsatellite markers. PLoS ONE 12: e0183384. 10.1371/journal.pone.0183384 PMC5576660 28854285

[nph71555-bib-0020] Huang T , Hodgens C , Prakash S , Marconi M , Wabnik K , Sozzani R , Wagner D . 2026. A negative feedback loop between TERMINAL FLOWER1 and LEAFY protects inflorescence indeterminacy. Science 391: eadv5429. 10.1126/science.adv5429 41570111

[nph71555-bib-0021] Hwang DY , Park S , Lee S , Lee SS , Imaizumi T , Song YH . 2019. GIGANTEA regulates the timing stabilization of CONSTANS by altering the interaction between FKF1 and ZEITLUPE. Molecules and Cells 42: 693–701. 10.14348/molcells.2019.0199 PMC6821452 31617339

[nph71555-bib-0022] Hyun Y , Vincent C , Tilmes V , Bergonzi S , Kiefer C , Richter R , Martinez‐Gallegos R , Severing E , Coupland G . 2019. A regulatory circuit conferring varied flowering response to cold in annual and perennial plants. Science 363: 409–412. 10.1126/science.aau8197 30679374

[nph71555-bib-0023] Imaizumi T , Tran HG , Swartz TE , Briggs WR , Kay SA . 2003. FKF1 is essential for photoperiodic‐specific light signalling in Arabidopsis. Nature 426: 302–306. 10.1038/nature02090 14628054

[nph71555-bib-0024] Ishikawa R , Aoki M , Kurotani K‐i , Yokoi S , Shinomura T , Takano M , Shimamoto K . 2011. Phytochrome B regulates Heading date 1 (Hd1)‐mediated expression of rice florigen Hd3a and critical day length in rice. Molecular Genetics and Genomics 285: 461–470. 10.1007/s00438-011-0621-4 21512732

[nph71555-bib-0025] Karimi M , Inzé D , Depicker A . 2002. GATEWAY™ vectors for Agrobacterium‐mediated plant transformation. Trends in Plant Science 7: 193–195. 10.1016/s1360-1385(02)02251-3 11992820

[nph71555-bib-0026] Kobayashi Y , Kaya H , Goto K , Iwabuchi M , Araki T . 1999. A pair of related genes with antagonistic roles in mediating flowering signals. Science 286: 1960–1962. 10.1126/science.286.5446.1960 10583960

[nph71555-bib-0027] Komiya R , Yokoi S , Shimamoto K . 2009. A gene network for long‐day flowering activates RFT1 encoding a mobile flowering signal in rice. Development 136: 3443–3450. 10.1242/dev.040170 19762423

[nph71555-bib-0028] Kong F , Liu B , Xia Z , Sato S , Kim BM , Watanabe S , Yamada T , Tabata S , Kanazawa A , Harada K . 2010. Two coordinately regulated homologs of FLOWERING LOCUS T are involved in the control of photoperiodic flowering in soybean. Plant Physiology 154: 1220–1231. 10.1104/pp.110.160796 PMC2971601 20864544

[nph71555-bib-0029] Koskela EA , Kurokura T , Toivainen T , Sønsteby A , Heide OM , Sargent DJ , Isobe S , Jaakola L , Hilmarsson H , Elomaa P . 2017. Altered regulation of TERMINAL FLOWER 1 causes the unique vernalisation response in an arctic woodland strawberry accession. New Phytologist 216: 841–853. 10.1111/nph.14734 28815698

[nph71555-bib-0030] Koskela EA , Mouhu K , Albani MC , Kurokura T , Rantanen M , Sargent DJ , Battey NH , Coupland G , Elomaa P , Hytönen T . 2012. Mutation in TERMINAL FLOWER1 reverses the photoperiodic requirement for flowering in the wild strawberry Fragaria vesca. Plant Physiology 159: 1043–1054. 10.1104/pp.112.196659 PMC3387692 22566495

[nph71555-bib-0031] Kurokura T , Samad S , Koskela E , Mouhu K , Hytönen T . 2017. Fragaria vesca CONSTANS controls photoperiodic flowering and vegetative development. Journal of Experimental Botany 68: 4839–4850. 10.1093/jxb/erx301 PMC5853477 29048562

[nph71555-bib-0032] Lazaro A , Obeng‐Hinneh E , Albani MC . 2018. Extended vernalization regulates inflorescence fate in *Arabis alpina* by stably silencing PERPETUAL FLOWERING1. Plant Physiology 176: 2819–2833. 10.1104/pp.17.01754 PMC5884582 29467177

[nph71555-bib-0033] Ledger S , Strayer C , Ashton F , Kay SA , Putterill J . 2001. Analysis of the function of two circadian‐regulated CONSTANS‐LIKE genes. The Plant Journal 26: 15–22. 10.1046/j.1365-313x.2001.01003.x 11359606

[nph71555-bib-0034] Lembinen S , Cieslak M , Zhang T , Mackenzie K , Elomaa P , Prusinkiewicz P , Hytönen T . 2023. Diversity of woodland strawberry inflorescences arises from heterochrony regulated by TERMINAL FLOWER 1 and FLOWERING LOCUS T. Plant Cell 35: 2079–2094. 10.1093/plcell/koad086 PMC10263268 36943776

[nph71555-bib-0035] Li F , Zhang X , Hu R , Wu F , Ma J , Meng Y , Fu Y . 2013. Identification and molecular characterization of FKF1 and GI homologous genes in soybean. PLoS ONE 8: e79036. 10.1371/journal.pone.0079036 PMC3827303 24236086

[nph71555-bib-0036] Liu L , Adrian J , Pankin A , Hu J , Dong X , Von Korff M , Turck F . 2014. Induced and natural variation of promoter length modulates the photoperiodic response of FLOWERING LOCUS T. Nature Communications 5: 4558. 10.1038/ncomms5558 PMC4143923 25087553

[nph71555-bib-0037] Mouhu K , Hytönen T , Folta K , Rantanen M , Paulin L , Auvinen P , Elomaa P . 2009. Identification of flowering genes in strawberry, a perennial SD plant. BMC Plant Biology 9: 122. 10.1186/1471-2229-9-122 PMC2761920 19785732

[nph71555-bib-0038] Mouhu K , Kurokura T , Koskela EA , Albert VA , Elomaa P , Hytönen T . 2013. The Fragaria vesca homolog of SUPPRESSOR OF OVEREXPRESSION OF CONSTANS1 represses flowering and promotes vegetative growth. Plant Cell 25: 3296–3310. 10.1105/tpc.113.115055 PMC3809533 24038650

[nph71555-bib-0039] Oosumi T , Gruszewski HA , Blischak LA , Baxter AJ , Wadl PA , Shuman JL , Veilleux RE , Shulaev V . 2006. High‐efficiency transformation of the diploid strawberry (*Fragaria vesca*) for functional genomics. Planta 223: 1219–1230. 10.1007/s00425-005-0170-3 16320068

[nph71555-bib-0040] Poppenwimer T , Mayrose I , DeMalach N . 2023. Revising the global biogeography of annual and perennial plants. Nature 624: 109–114. 10.1038/s41586-023-06644-x PMC10830411 37938778

[nph71555-bib-0065] R Core Team . 2023. R: a language and environment for statistical computing (Version 4.3.0). Vienna, Austria: R Foundation for Statistical Computing. [WWW document] URL https://www.r-project.org/.

[nph71555-bib-0041] Rantanen M , Kurokura T , Jiang P , Mouhu K , Hytönen T . 2015. Strawberry homologue of TERMINAL FLOWER 1 integrates photoperiod and temperature signals to inhibit flowering. The Plant Journal 82: 163–173. 10.1111/tpj.12809 25720985

[nph71555-bib-0042] Rantanen M , Kurokura T , Mouhu K , Pinho P , Tetri E , Halonen L , Palonen P , Elomaa P , Hytönen T . 2014. Light quality regulates flowering in FvFT1/FvTFL1 dependent manner in the woodland strawberry *Fragaria vesca* . Frontiers in Plant Science 5: 271. 10.3389/fpls.2014.00271 PMC4052200 24966865

[nph71555-bib-0043] Robinson MD , McCarthy DJ , Smyth GK . 2010. edgeR: a Bioconductor package for differential expression analysis of digital gene expression data. Bioinformatics 26: 139–140. 10.1093/bioinformatics/btp616 PMC2796818 19910308

[nph71555-bib-0044] Roggen A , Lloret A , Miotto Y , Wang K , Luxa K , Oruganti V , Della Pina S , van Driel AD , Hyun Y , Huettel B . 2025. The MIR157–SPL15 module regulates flowering and inflorescence development in *Arabidopsis thaliana* under short days and in *Arabis alpina* . PLoS Genetics 21: e1011799. 10.1371/journal.pgen.1011799 PMC12404414 40892775

[nph71555-bib-0045] Rosas U , Mei Y , Xie Q , Banta JA , Zhou RW , Seufferheld G , Gerard S , Chou L , Bhambhra N , Parks JD . 2014. Variation in Arabidopsis flowering time associated with *cis*‐regulatory variation in CONSTANS. Nature Communications 5: 3651. 10.1038/ncomms4651 PMC3997802 24736505

[nph71555-bib-0046] Samad S , Kurokura T , Koskela E , Toivainen T , Patel V , Mouhu K , Sargent DJ , Hytönen T . 2017. Additive QTLs on three chromosomes control flowering time in woodland strawberry (*Fragaria vesca* L.). Horticulture Research 4: 17020. 10.1038/hortres.2017.20 PMC5442962 28580150

[nph71555-bib-0066] Sasani S , Hemming MN , Oliver SN , Greenup A , Tavakkol‐Afshari R , Mahfoozi S , Poustini K , Sharifi H‐R , Dennis ES , Peacock WJ *et al*. 2009. The influence of vernalization and daylength on expression of flowering‐time genes in the shoot apex and leaves of barley (*Hordeum vulgare*). Journal of Experimental Botany 60: 2169–2178. 10.1093/jxb/erp098 PMC2682508 19357429

[nph71555-bib-0047] Sashidhar N , Coupland G . 2026. In perennial Arabis alpina, CONSTANS and FLOWERING LOCUS T have common and distinct effects on flowering and inflorescence architecture. New Phytologist 250: 970–987. 10.1111/nph.70994 PMC13000977 41656799

[nph71555-bib-0048] Sawa M , Nusinow DA , Kay SA , Imaizumi T . 2007. FKF1 and GIGANTEA complex formation is required for day‐length measurement in Arabidopsis. Science 318: 261–265. 10.1126/science.1146994 PMC3709017 17872410

[nph71555-bib-0049] Schnablová R , Huang L , Klimešová J , Šmarda P , Herben T . 2021. Inflorescence preformation prior to winter: a surprisingly widespread strategy that drives phenology of temperate perennial herbs. New Phytologist 229: 620–630. 10.1111/nph.16880 32805759

[nph71555-bib-0067] Song YH , Estrada DA , Johnson RS , Kim SK , Lee SY , MacCoss MJ , Imaizumi T . 2014. Distinct roles of FKF1, GIGANTEA, and ZEITLUPE proteins in the regulation of CONSTANS stability in *Arabidopsis* photoperiodic flowering. Proceedings of the National Academy of Sciences, USA 111: 17672–17677. 10.1073/pnas.1415375111 PMC4267339 25422419

[nph71555-bib-0050] Song YH , Shim JS , Kinmonth‐Schultz HA , Imaizumi T . 2015. Photoperiodic flowering: time measurement mechanisms in leaves. Annual Review of Plant Biology 66: 441–464. 10.1146/annurev-arplant-043014-115555 PMC4414745 25534513

[nph71555-bib-0051] Song YH , Smith RW , To BJ , Millar AJ , Imaizumi T . 2012. FKF1 conveys timing information for CONSTANS stabilization in photoperiodic flowering. Science 336: 1045–1049. 10.1126/science.1219644 PMC3737243 22628657

[nph71555-bib-0052] Tamaki S , Matsuo S , Wong HL , Yokoi S , Shimamoto K . 2007. Hd3a protein is a mobile flowering signal in rice. Science 316: 1033–1036. 10.1126/science.1141753 17446351

[nph71555-bib-0053] Toivainen T , Salonen JS , Kirshner J , Lembinen S , De Kort H , Lyyski A , Edger PP , Hilmarsson HS , Hallsson JH , Sargent DJ *et al*. 2026. The Late Quaternary climate impact on the genome of the woodland strawberry (*Fragaria vesca*), a perennial herb. Communications Biology 9: 263. 10.1038/s42003-026-09539-5 PMC12913768 41535390

[nph71555-bib-0054] Valverde F , Mouradov A , Soppe W , Ravenscroft D , Samach A , Coupland G . 2004. Photoreceptor regulation of CONSTANS protein in photoperiodic flowering. Science 303: 1003–1006. 10.1126/science.1091761 14963328

[nph71555-bib-0055] Vicentini G , Biancucci M , Mineri L , Chirivì D , Giaume F , Miao Y , Kyozuka J , Brambilla V , Betti C , Fornara F . 2023. Environmental control of rice flowering time. Plant Communications 4: 100610. 10.1016/j.xplc.2023.100610 PMC10504588 37147799

[nph71555-bib-0068] Wahl V , Ponnu J , Schlereth A , Arrivault S , Langenecker T , Franke A , Feil R , Lunn JE , Stitt M , Schmid M . 2013. Regulation of flowering by trehalose‐6‐phosphate signaling in *Arabidopsis thaliana* . Science 339: 704–707. 10.1126/science.1230406 23393265

[nph71555-bib-0056] Wang R , Albani MC , Vincent C , Bergonzi S , Luan M , Bai Y , Kiefer C , Castillo R , Coupland G . 2011. Aa TFL1 confers an age‐dependent response to vernalization in perennial *Arabis alpina* . Plant Cell 23: 1307–1321. 10.1105/tpc.111.083451 PMC3101554 21498681

[nph71555-bib-0057] Wang R , Farrona S , Vincent C , Joecker A , Schoof H , Turck F , Alonso‐Blanco C , Coupland G , Albani MC . 2009. PEP1 regulates perennial flowering in *Arabis alpina* . Nature 459: 423–427. 10.1038/nature07988 19369938

[nph71555-bib-0058] Wu F , Price BW , Haider W , Seufferheld G , Nelson R , Hanzawa Y . 2014. Functional and evolutionary characterization of the CONSTANS gene family in short‐day photoperiodic flowering in soybean. PLoS ONE 9: e85754. 10.1371/journal.pone.0085754 PMC3897488 24465684

[nph71555-bib-0059] Xia Z , Watanabe S , Yamada T , Tsubokura Y , Nakashima H , Zhai H , Anai T , Sato S , Yamazaki T , Lü S . 2012. Positional cloning and characterization reveal the molecular basis for soybean maturity locus E1 that regulates photoperiodic flowering. Proceedings of the National Academy of Sciences, USA 109: E2155–E2164. 10.1073/pnas.1117982109 PMC3420212 22619331

[nph71555-bib-0060] Xu M , Yamagishi N , Zhao C , Takeshima R , Kasai M , Watanabe S , Kanazawa A , Yoshikawa N , Liu B , Yamada T . 2015. The soybean‐specific maturity gene E1 family of floral repressors controls night‐break responses through down‐regulation of FLOWERING LOCUS T orthologs. Plant Physiology 168: 1735–1746. 10.1104/pp.15.00763 PMC4528769 26134161

[nph71555-bib-0061] Zhang Y , Kan L , Hu S , Liu Z , Kang C . 2023. Roles and evolution of four LEAFY homologs in floral patterning and leaf development in woodland strawberry. Plant Physiology 192: 240–255. 10.1093/plphys/kiad067 PMC10152680 36732676

[nph71555-bib-0062] Zhu Y , Klasfeld S , Jeong CW , Jin R , Goto K , Yamaguchi N , Wagner D . 2020. TERMINAL FLOWER 1‐FD complex target genes and competition with FLOWERING LOCUS T. Nature Communications 11: 5118. 10.1038/s41467-020-18782-1 PMC7550357 33046692

[nph71555-bib-0063] Zong W , Ren D , Huang M , Sun K , Feng J , Zhao J , Xiao D , Xie W , Liu S , Zhang H . 2021. Strong photoperiod sensitivity is controlled by cooperation and competition among Hd1, Ghd7 and DTH8 in rice heading. New Phytologist 229: 1635–1649. 10.1111/nph.16946 PMC7821112 33089895

