# Supplementary material for: FvGI–FvCO–FvFT1 pathway activates FvTFL1 in long days to repress flowering in woodland strawberry (Fragaria vesca)
Source: New Phytol. 2026 Aug 28;252(3):1269–82. doi: 10.1111/nph.71555 (PMC13628546; doi:10.1111/nph.71555)
Supplement: Supplementary file 1 [file NPH-252-1269-s003.pdf]

New Phytologist

New Phytologist **Supporting Information**

Article title: ***FvTFL1* reverses the function of *FvGI-FvCO-FvFT1* pathway in the photoperiodic flowering of woodland strawberry**

Authors: Quan Zhou, Sergei Lembinen, Tuomas Toivainen, Takeshi Kurokura, Guangxun Fan, Paula Elomaa, Elli Koskela, Timo Hytönen

Article acceptance date: 13 August 2026

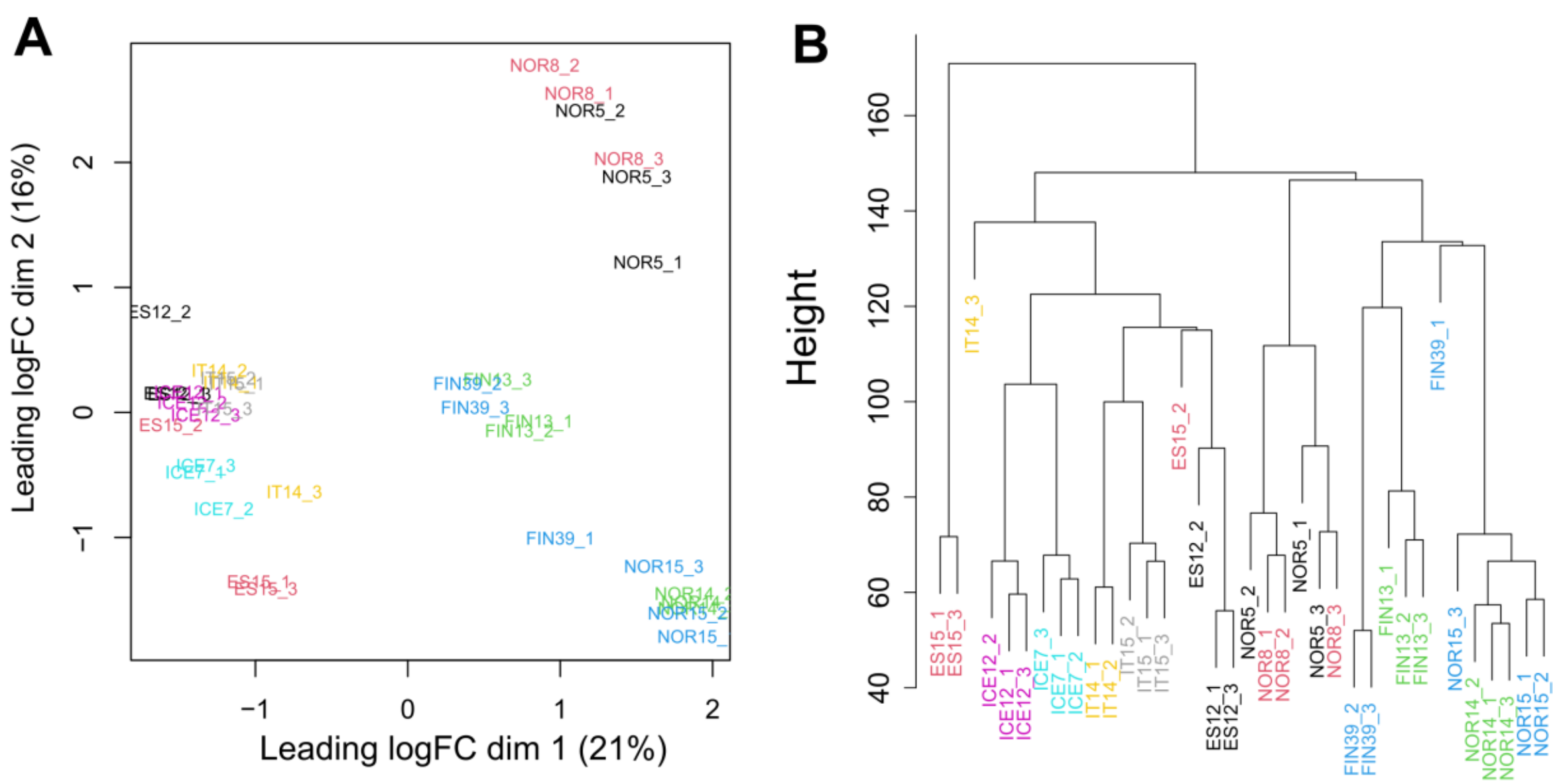

Fig. S1. Clustering of RNA sequencing samples. Multidimensional scaling (MDS) plot (A) and a dendrogram (B) showing clustering of RNA sequencing samples. Leaf samples of six eastern and six western accessions of *Fragaria vesca* collected at ZT4 under 18-h long days were analysed.

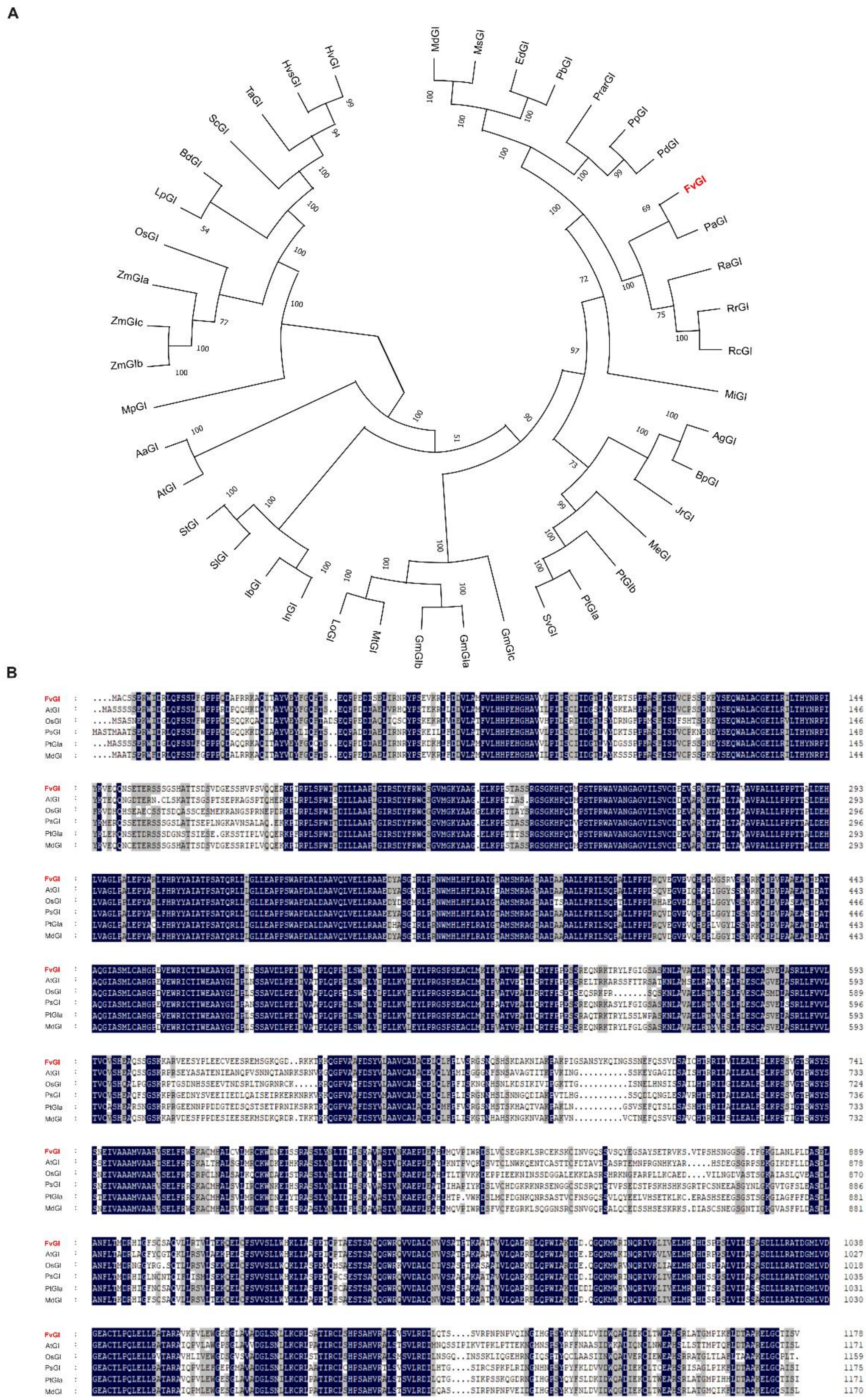
A
HvGI
HvsGI
TaGI
ScGI
BdGI
LpGI
OsGI
ZmGIa
ZmGIc
ZmGIb
MpGI
AaGI
AtGI
StGI
SlGI
IbGI
InGI
LoGI
MtGI
GmGIb
GmGIa
GmGIc
SvGI
PtGIa
PtGIb
MeGI
JrGI
BpGI
AgGI
MiGI
RcGI
RrGI
RaGI
PaGI
FvGI
PdGI
PpGI
PrarGI
PbGI
EdGI
MsGI
MdGI
B
FvGI
AtGI
OsGI
PsGI
PtGIa
MdGI

Fig. S2. Phylogenetic analysis and multiple sequence alignment of GI homologs. (A) Phylogenetic analysis of GI proteins among different species. Numbers at the nodes represent bootstrap values. (B) Multiple sequence alignment of full-length GI protein sequences from species in which GI function has been characterized. Conserved amino acid residues are highlighted according to the degree of sequence conservation: black shading indicates positions with 100% conservation, grey represents ≥75% conservation, and white denotes ≤75% conservation. Species abbreviations: *Aa, Arabis alpina; Ag, Alnus glutinosa; At, Arabidopsis thaliana; Bd, Brachypodium distachyon; Bp, Betula platyphylla; Ed, Eriobotrya deflexa; Fv, Fragaria vesca; Gm, Glycine max; Hv, Hordeum vulgare; Ib, Ipomoea batatas; In, Ipomoea nil; Jr, Juglans regia; Lo, Lathyrus oleraceus; Lp, Lolium perenne; Md, Malus domestica; Me, Manihot esculenta; Mi, Mangifera indica; Mp, Marchantia polymorpha; Ms, Malus sylvestris; Mt, Medigago truncatula; Os, Oryza sativa; Pa, Potentilla anserina; Pb, Pyrus x bretschneideri; Pd, Prunus dulcis; Pp, Prunus persica; Pt, Populus trichocarpa; Ra, Rubus arcutus; Rc, Rosa chinensis; Rr, Rosa rugosa; Sc, Secale cereale; Sl, Solanum lycopersicum; St, Solanum tuberosum; Sv, Salix viminalis; Zm, Ta, Triticum aestivum; Zea mays.* Details of protein sequences are found in Table S4.

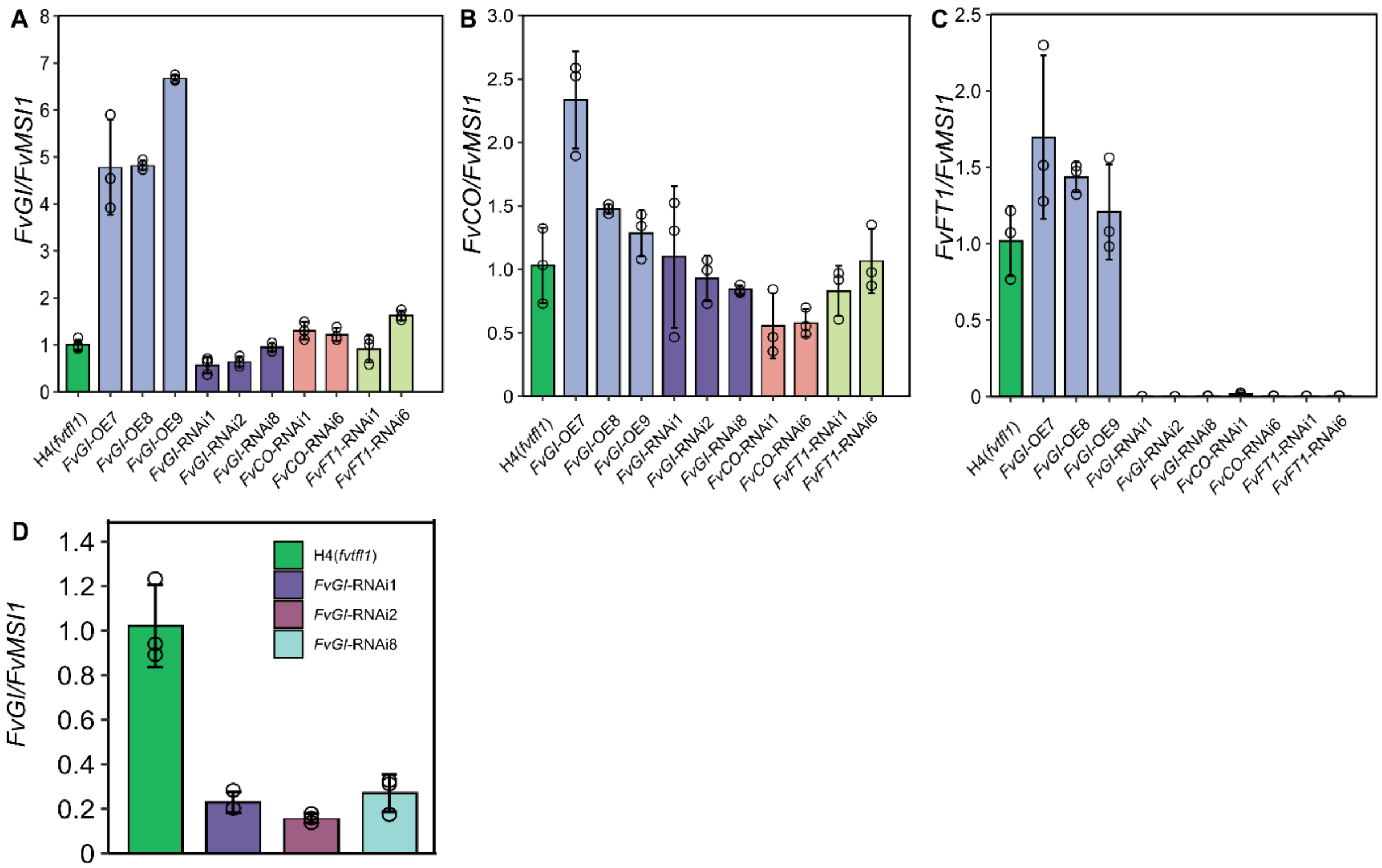

Fig. S3. Gene expression in *Fragaria vesca* accession H4 and transgenic lines. (A-C) RT-qPCR analysis *FvGI* (A), *FvCO* (B) and *FvFT1* (C) expression in leaf samples of H4, *FvGI*-OE lines and RNAi silencing lines for *FvGI*, *FvCO*, and *FvFT1*. Leaf samples including one leaflet per biological replicate (n = 3) were collected at zeitgeber time 4 (ZT4) under 18-h long days (LD^{18h}). Values are mean ± SD. (D) RT-qPCR analysis of *FvGI* in H4 and indicated RNAi lines. Leaf samples including one leaflet per biological replicate (n = 3) were collected at ZT8 under LD^{18h} conditions. Values are mean ± SD.

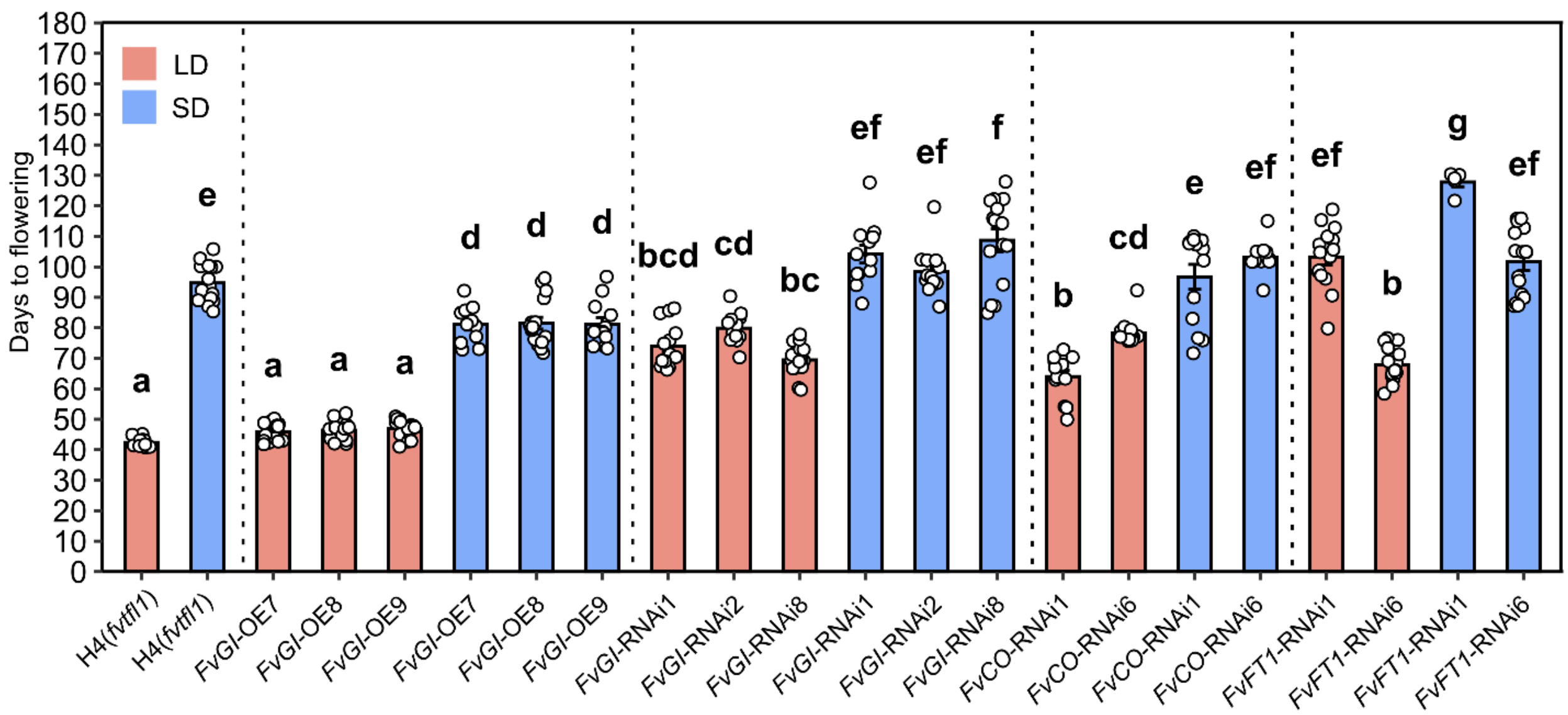

Fig. S4. Flowering time of *Fragaria vesca* accession H4 and indicated transgenic lines. Plants were grown under 18-h long days (LD) or 12-h short days (SD) conditions. Values are mean ± SD, n = 11-16. Different letters indicate statistically significant differences as determined by two-way ANOVA followed by Tukey's HSD multiple comparisons test ($P < 0.05$).

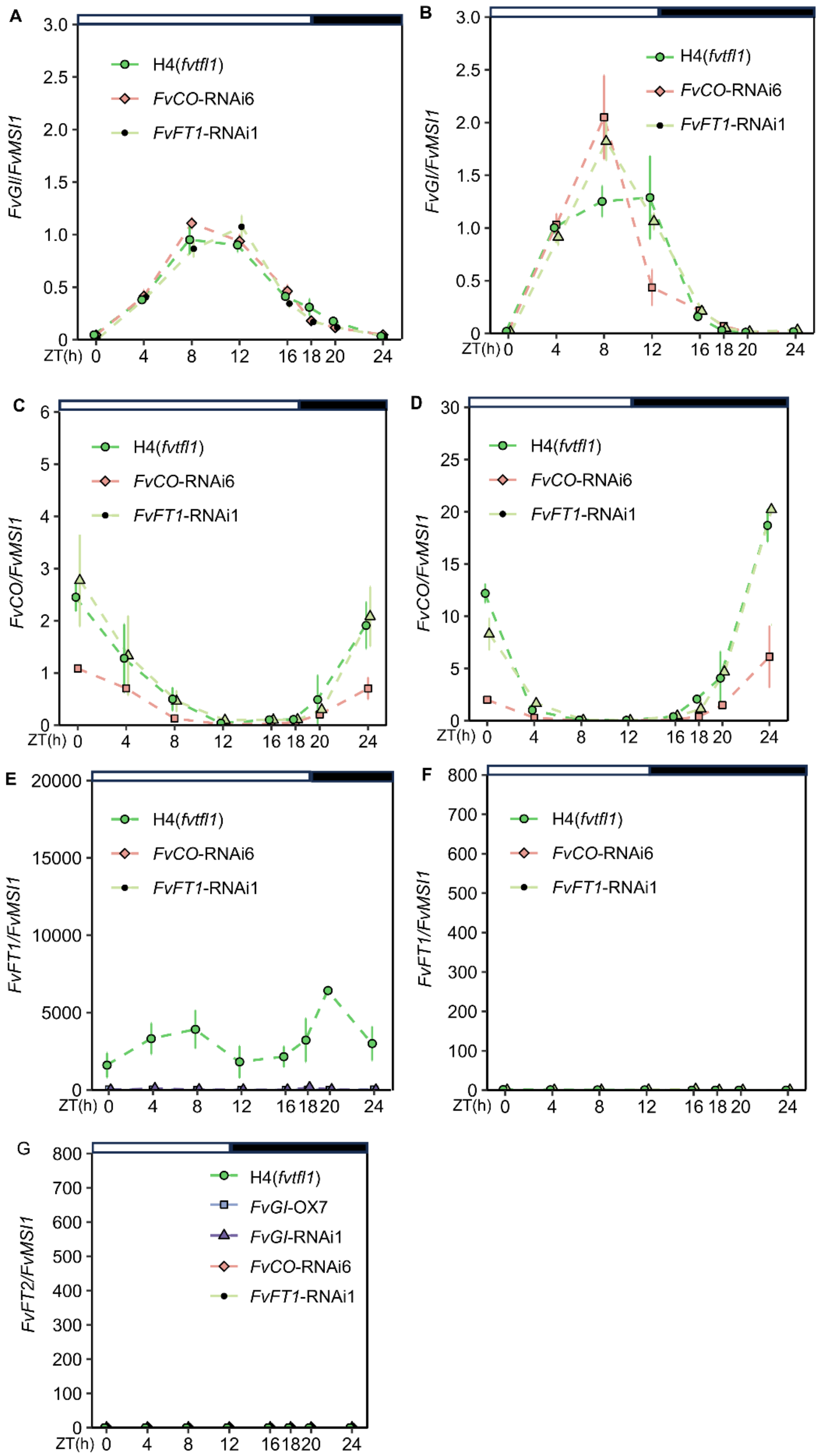
A
B
C
D
E
F
G
FvGI/FvMSI1
FvCO/FvMSI1
FvFT1/FvMSI1
FvFT2/FvMSI1
ZT(h)
H4(fvtfl1)
FvCO-RNAi6
FvFT1-RNAi1
FvGI-OX7
FvGI-RNAi1

Fig. S5. Diurnal gene expression rhythms in *Fragaria vesca* accession H4 and transgenic lines. (A-F) Diurnal expression rhythms of *FvGI* (A, B), *FvCO* (C, D) and *FvFT1* (E, F) in leaves of H4, and *FvCO*- and *FvFT1*-RNAi lines grown under 18-h long days (left) and 12-h short days (SD^{12h}, right) conditions. (G) Diurnal gene expression analysis of *FvFT2* in leaf samples of H4 and its *FvGI*-OE and *FvGI*-, *FvCO*- and *FvFT1*- silencing lines grown under SD^{12h} conditions. Values are mean ± SD (n = 3). White and black bars above the panels represent light and dark periods, respectively. Zeitgeber time 0 (ZT0) = lights on. Expression levels were measured in leaves after 3 weeks of light treatments using RT-qPCR.

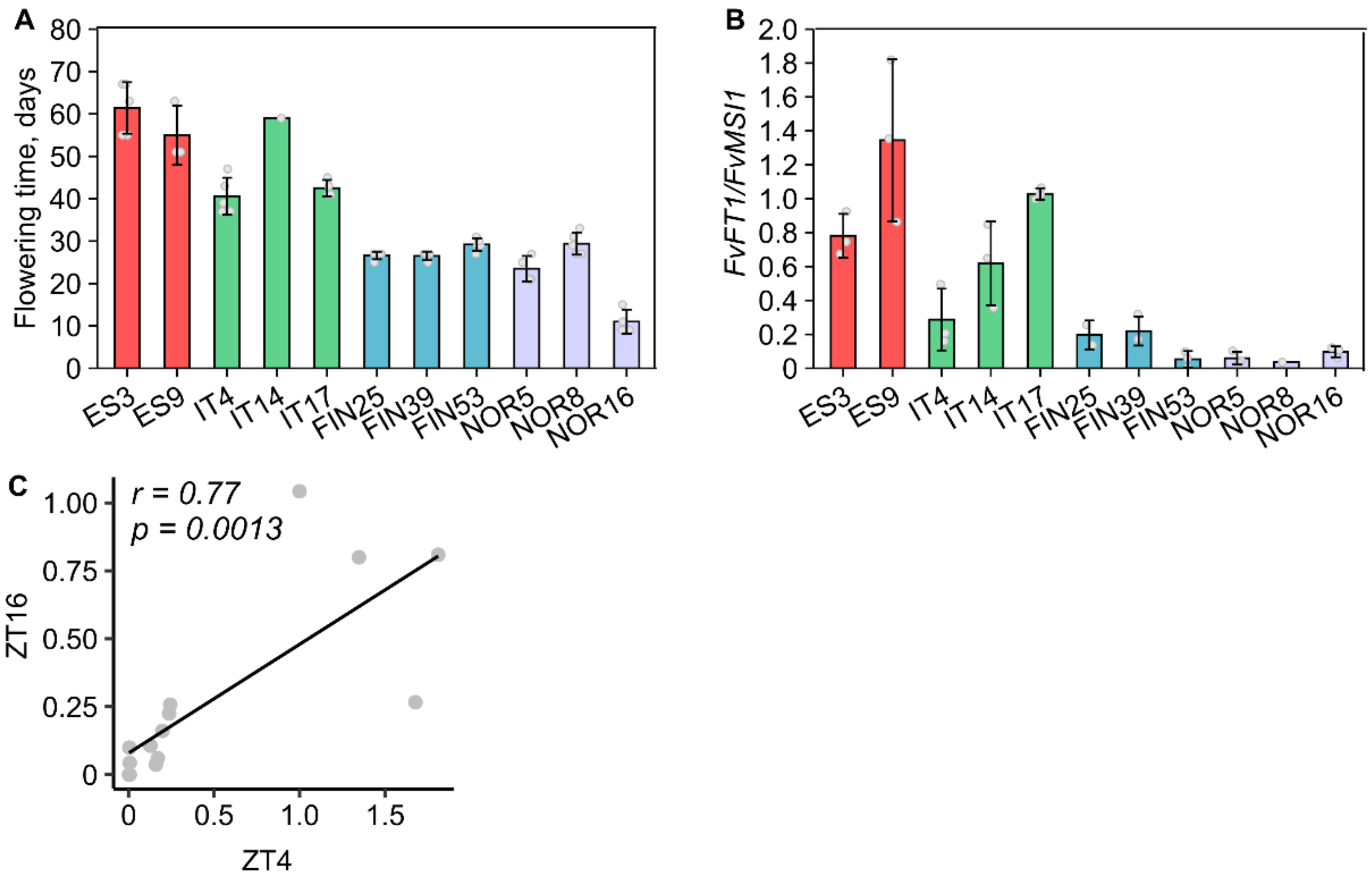

Fig. S6. Flowering time and *FvFT1* expression in natural accessions of *Fragaria vesca*. (A, B) Flowering time (A) and *FvFT1* expression levels (B) of indicated accessions. In A, plants (n = 5) were induced to flower under 12-h short day conditions (6 weeks), followed by flowering time observations under 18-h long days (LD^{18h}). In B, leaf samples (n = 3) were collected at zeitgeber time 16 (ZT16) under LD^{18h} conditions. In A and B, values are mean ± SD. (C) Pearson's correlation between the relative expression of *FvFT1* in leaves collected from ZT4 (data from Figure 5e) and ZT16 among 13 natural accessions of *Fragaria vesca*.

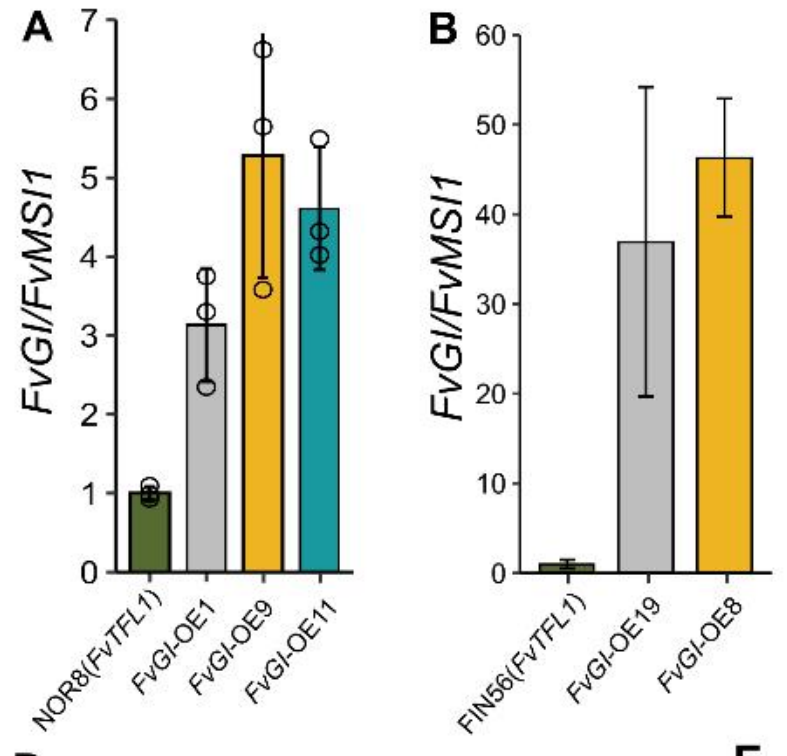

C

Genotype	Photoperiod (light/darkness)	Number of plants	Flowering plants (%)	Days to anthesis ± SD	Leaves to anthesis ± SD
NOR8(*FvTFL1*)	SD 12/12	10	100	28.40 ± 1.83	9.10 ± 0.73
	LD 18/6	10	0	>100	>18
FvGI-OX1	SD 12/12	10	40	36.25 ± 6.50	10.25 ± 0.50
	LD 18/6	10	0	>100	>18
FvGI-OX9	SD 12/12	10	0	>100	>18
	LD 18/6	10	0	>100	>18
FvGI-OX11	SD 12/12	10	0	>100	>18
	LD 18/6	10	0	>100	>18

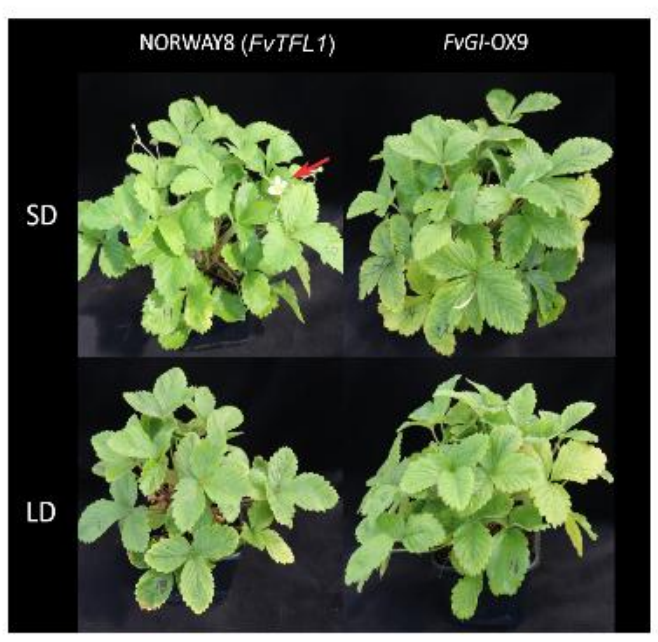

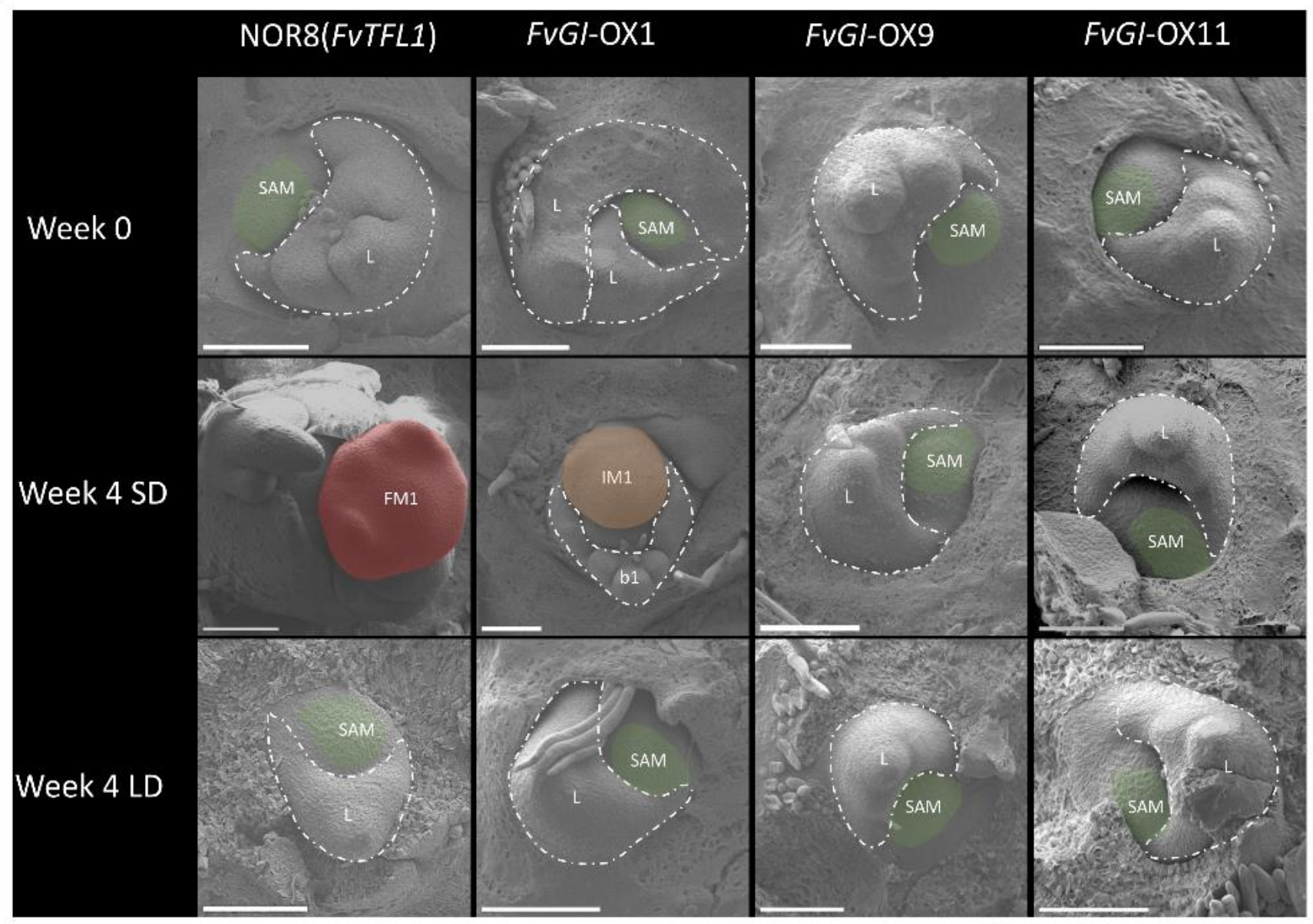

F

Genotpye	Photoperiod	Number of Plants	Flowering percentage(%)	Leaves to anthesis (± sd)
FIN56(*FvTFL1*)	SD (12h/12h)	9	100	13.6±0.
FvGI-OE8	SD (12h/12h)	14	28.6	16.3±2.2
FvGI-OE19	SD (12h/12h)	14	0	NA

Fig. S7. Gene expression and phenotypes of indicated *Fragaria vesca* genotypes. (A, B) RT-qPCR analysis *FvGI* in NOR8 (A) and FIN56 (B) accessions and indicated *FvGI* overexpression (OE) lines in shoot apex samples. For each biological replicate (n = 3), three shoot apices were harvested under 18-h long day (LD^{18h}) conditions. Values are mean ± SD. (C) Flowering phenotypes of NOR8 and *FvGI*-OE lines. Flowering time is indicated as days to flowering and leaf numbers to flowering after indicated treatments of four weeks. After treatments, plants were transferred to LD^{18h} for flowering time observations for up to 12 weeks. (D) Phenotypes of indicated treatments after photoperiodic treatments. Red arrow shows flower in NOR8 after 12-h short day (SD^{12h}) treatment. (E) Scanning electron microscope images of meristems at week 0 and after 4 weeks under LD^{18h} and SD^{12h} treatments. All meristems remained vegetative under LD^{18h} treatment. Under SD^{12h} treatment, NOR8 displayed early stage of flower meristem (FM1), whereas a weaker *FvGI*-OE line had reached inflorescence meristem stage (IM1). Meristems of two strong *FvGI*-OE lines remained vegetative. White dashes: leaves. Green indicated area: vegetative shoot apical

meristem. Orange indicated area: inflorescence meristem (IM1). Red area: flower meristem (FM1). Bars in all images indicate 100 μm. (F) Flowering time of FIN56 and its *FvGI*-OE lines. Plants were grown under SD^{12h} conditions for five weeks, followed by flowering time observations under LD^{18h}.

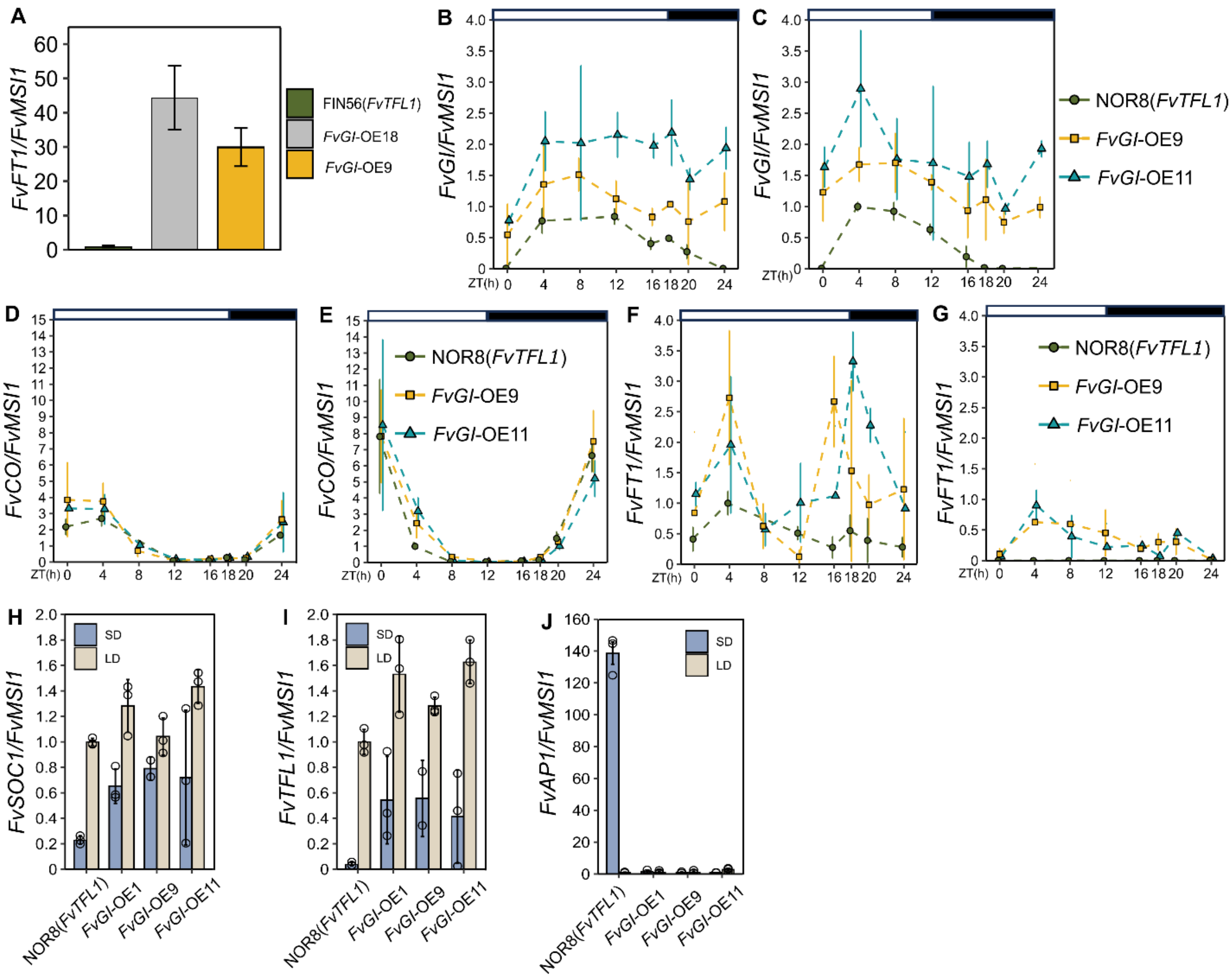

Fig. S8. Expression of flowering time genes in *Fragaria vesca* accession NOR8 and *FvGI* overexpression lines. (A) RT-qPCR analysis *FvFT1* in FIN56 and *FvGI* overexpression (*FvGI*-OE) lines in leaf samples collected at zeitgeber time 4 (ZT4) under 18-h long day (LD^{18h}) conditions. (B-G) The expression of *FvGI* (B, C), *FvCO* (D, E) and *FvFT1* (F, G) over 24 h diurnal cycle in NOR8 and *FvGI*-OE lines grown under LD^{18h} (B, D, F) and 12-h short days (SD^{12h}) (C, E, G) for four weeks. Values are mean ± SD (n = 3). (H-J) RT-qPCR analysis *FvSOC1* (H), *FvTFL1* (I) and *FvAP1* (J) in shoot apex samples. Three shoot apices were harvested for each biological replicate after 4 weeks under indicated treatments. Values are mean ± SD (n = 3).

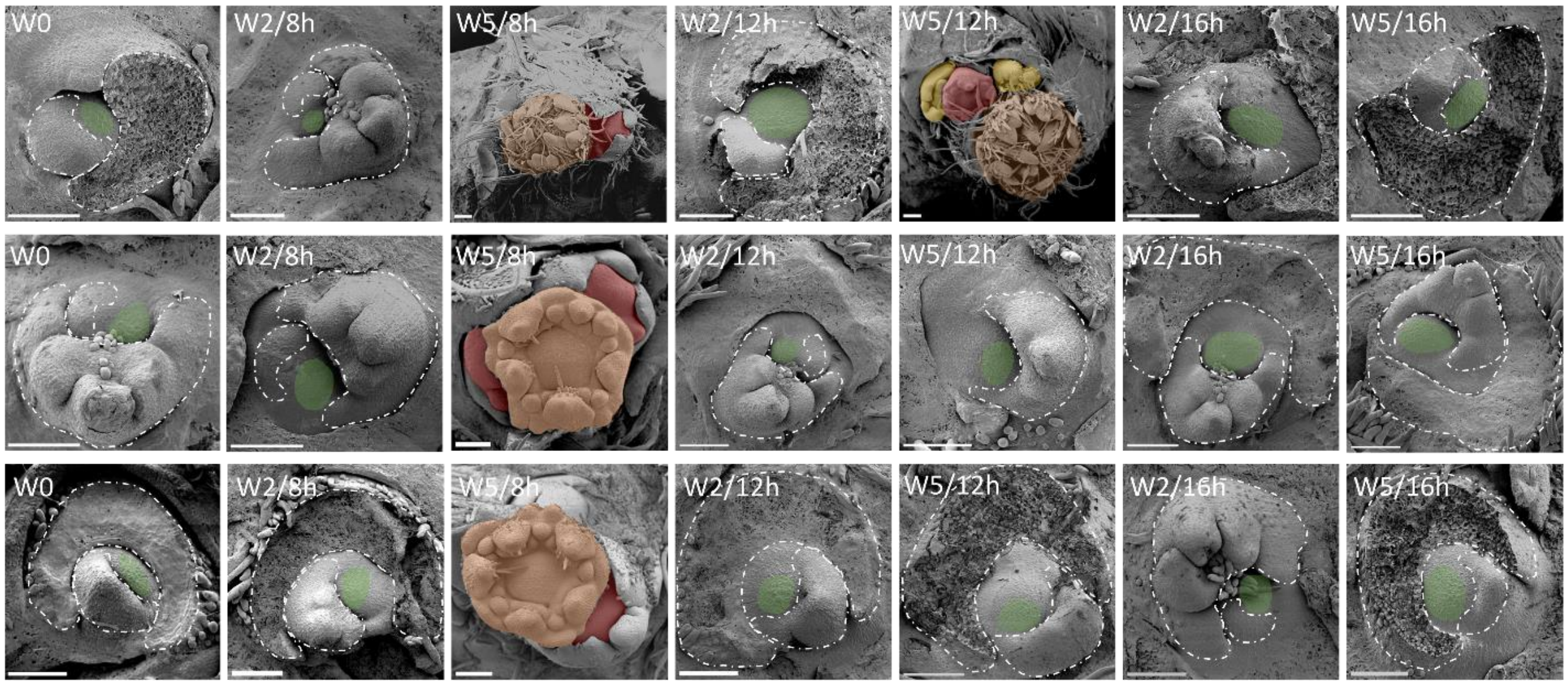

Fig. S9. Scanning electron microscope images of *Fragaria vesca* meristems. Shoot apical meristems (SAM) of NOR8 (row 1), and *FvGI* overexpression lines *FvGI*-OE9 (row 2) and *FvGI*-OE11 (row 3) after 0 (W0), 2 (W2) and 5 weeks (W5) under 8-h, 12-h, and 16-h photoperiods. After five weeks under 8-h photoperiod, NOR8 plants displayed late stage of flower meristem 1 (FM1, orange) and initiation of FM2 (red), whereas *FvGI*-OE plants exhibited an earlier FM1 (orange) stage with one or two visible later inflorescence meristems (IM2, red). After five weeks under 12-h photoperiod, more advanced developmental stage of NOR8 was found compared with NOR8 under 8-h photoperiod, with visible FM1 (orange), FM2 (red) and two lateral IM3 (yellow) in the shoot apex. Vegetative SAMs (green) were found in *FvGI*-OE plants under 12-h photoperiod and in all plants under 16-h photoperiod. White dashes area indicates leaves. Scale bars in all images are equal to 100 μm.

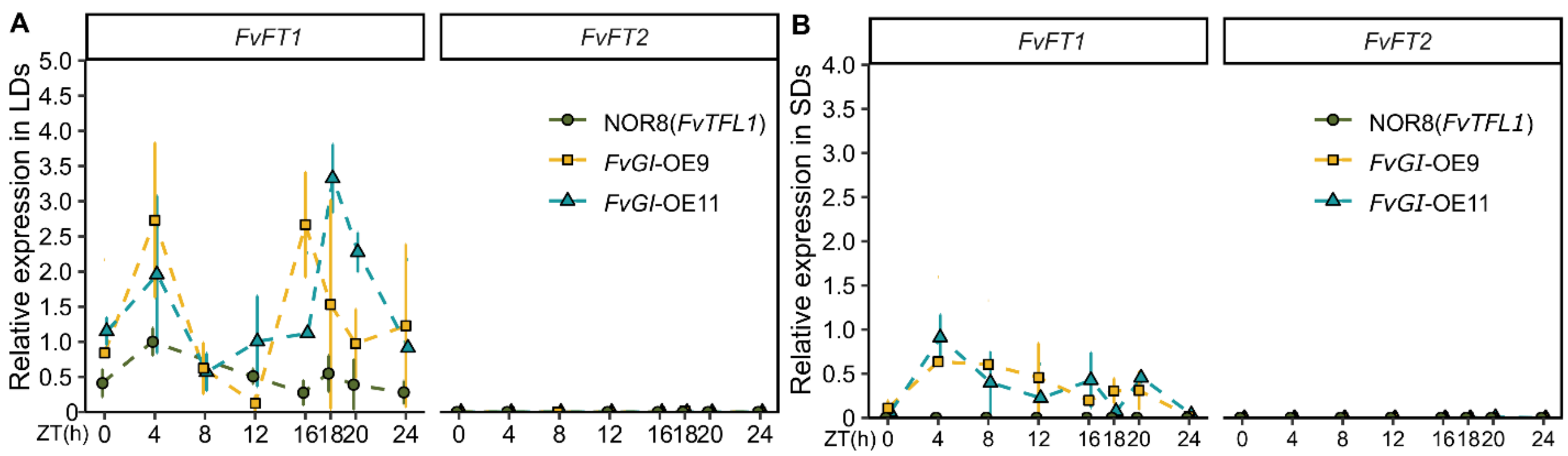

Fig. S10. Expression of *FvFT1* and *FvFT2* in *Fragaria vesca* accession NOR8 and *FvGI* overexpression lines. (A, B) Gene expression over 24-h cycle was analyzed in leaves of NOR8 and its *FvGI* overexpression (*FvGI*-OE) lines harvested under 18-h long day (LD^{18h}) (A) and 12-h short day (SD^{12h}) (B) conditions. Values are mean ± SD (n = 3).

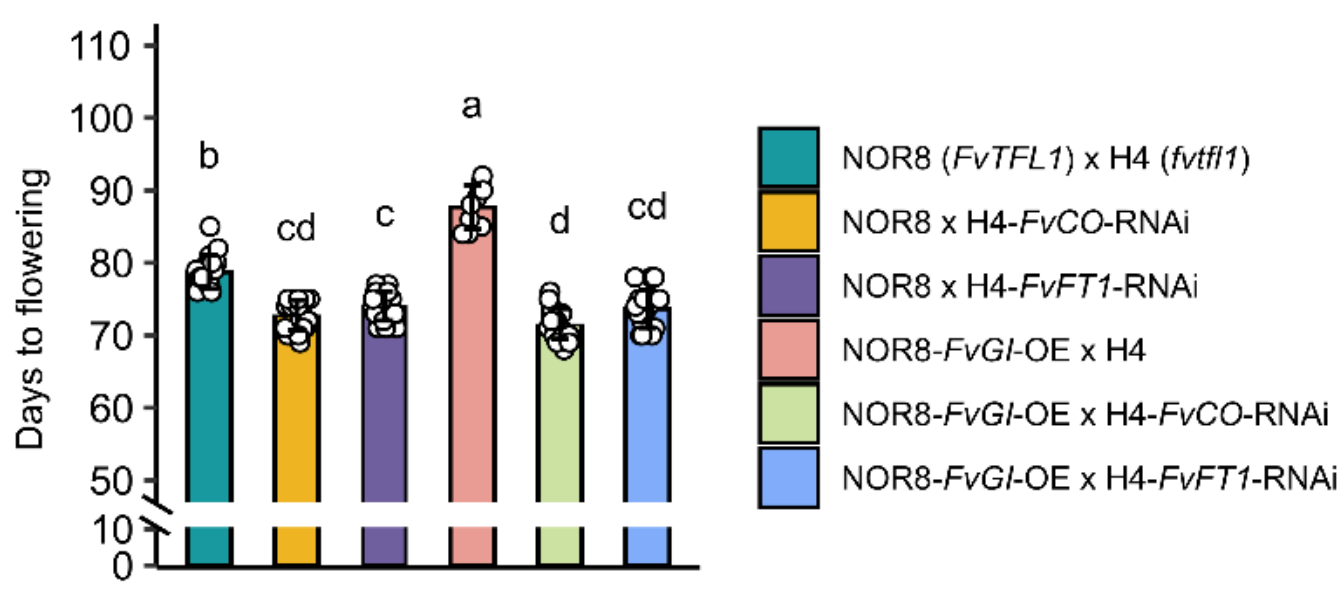

Fig. S11. Flowering time of indicated *Fragaria vesca* hybrids. Four-week-old plants, clonally propagated from hybrid seedlings were subjected to 12-h short days at 17°C for eight weeks followed by phenotyping under 18-h long days. OE = overexpression. Values are mean ± SD (n = 18). Different letters indicate statistically significant differences as determined by one-way ANOVA followed by Tukey's HSD multiple comparisons test ($P < 0.05$).

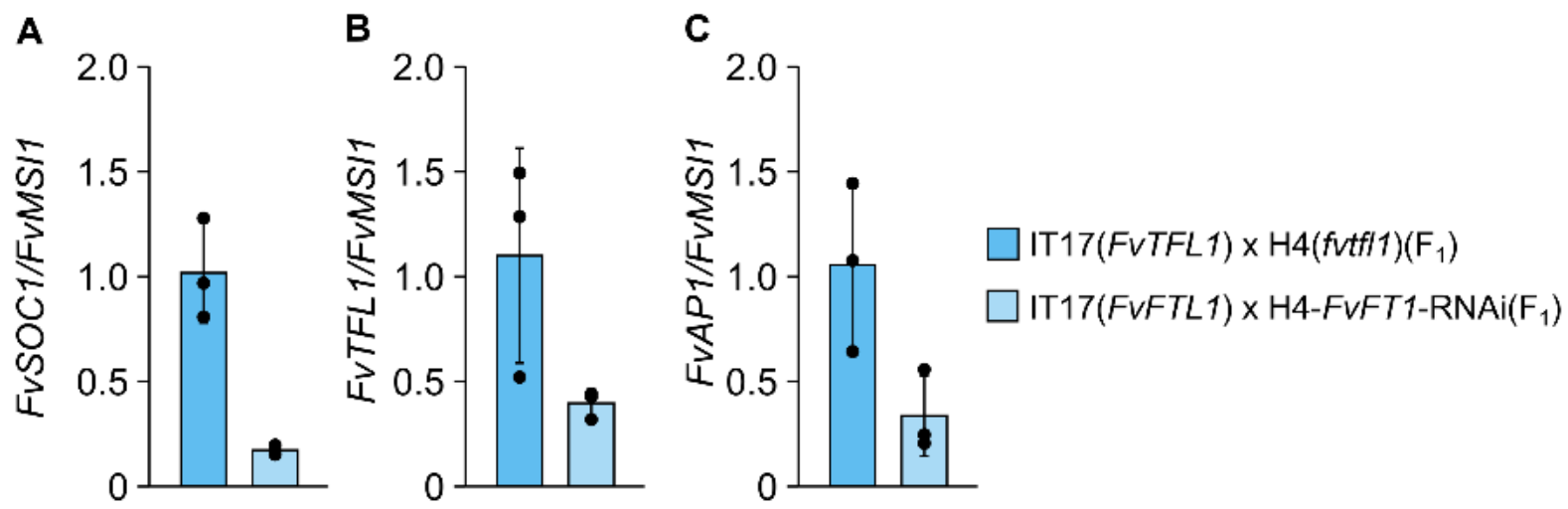

Fig. S12. Expression of flowering genes in indicated *Fragaria vesca* hybrids. (A-C) The expression of *FvSOC1* (A), *FvTFL1* (B) and *FvAP1* (C) in shoot apices harvested under 18-h long day conditions in IT17 x H4 and IT17 x H4 *FvFT1*-RNAi F1 plants. Three shoot apices were harvested for each biological replicate. Values are mean ± SD (n = 3).

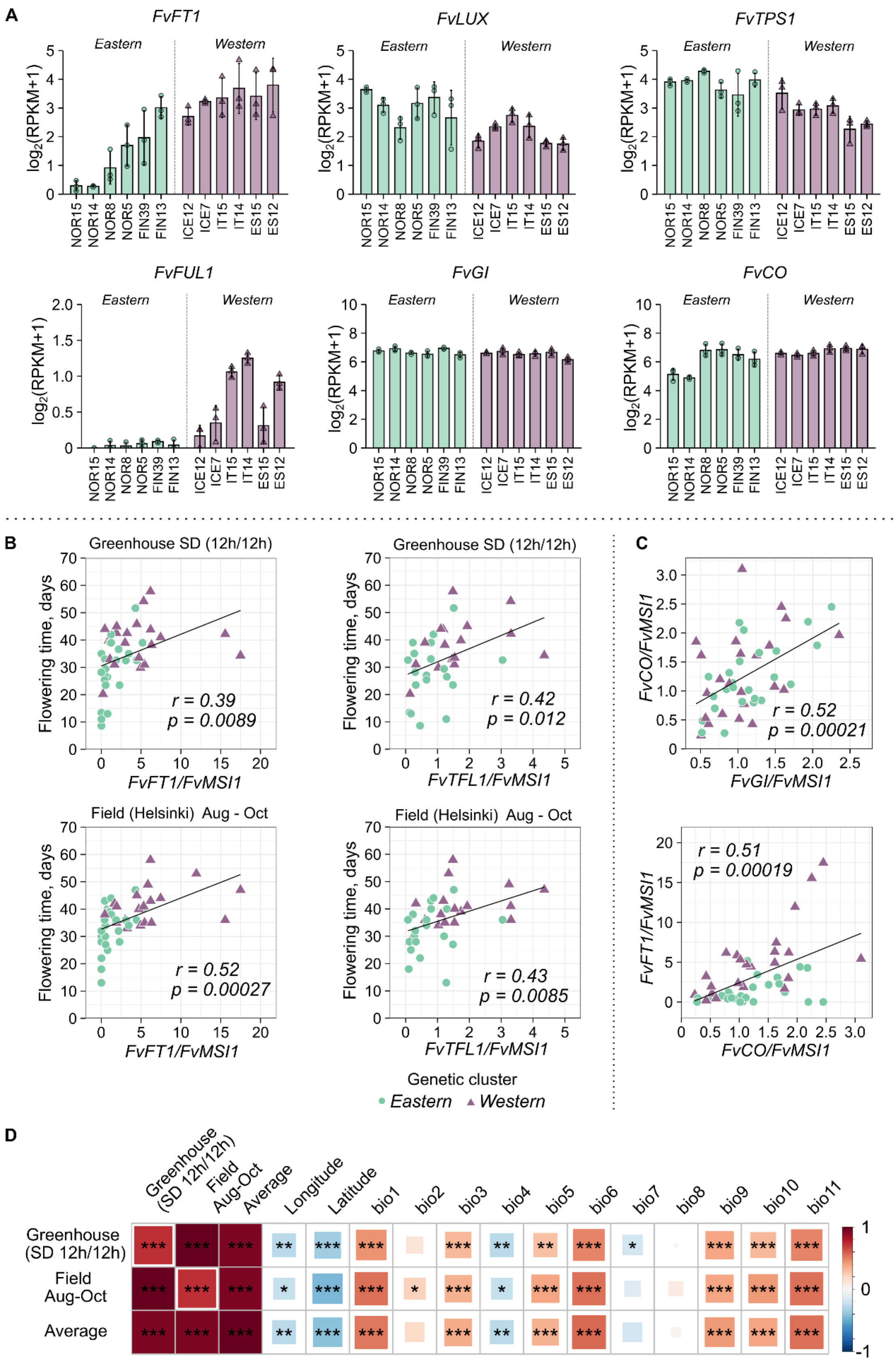
A
FvFT1
FvLUX
FvTPS1
FvFUL1
FvGI
FvCO
Eastern
Western
log2(RPKM+1)
NOR15
NOR14
NOR8
NOR5
FIN39
FIN13
ICE12
ICE7
IT15
IT14
ES15
ES12
B
Greenhouse SD (12h/12h)
Field (Helsinki) Aug - Oct
Flowering time, days
FvFT1/FvMSI1
FvTFL1/FvMSI1
r = 0.39
p = 0.0089
r = 0.42
p = 0.012
r = 0.52
p = 0.00027
r = 0.43
p = 0.0085
C
FvCO/FvMSI1
FvGI/FvMSI1
r = 0.52
p = 0.00021
FvFT1/FvMSI1
FvCO/FvMSI1
r = 0.51
p = 0.00019
Genetic cluster
Eastern
Western
D
Greenhouse (SD 12h/12h)
Field Aug-Oct
Average
Longitude
Latitude
bio1
bio2
bio3
bio4
bio5
bio6
bio7
bio8
bio9
bio10
bio11

Fig. S13. Comparison of gene expression levels in *Fragaria vesca* accessions and correlations between gene expression, flowering time, and bioclimatic and geographical variables. (A) Expression levels of *FvFT1*, *FvLUX*, *FvTPS1*, *FvFUL1*, *FvGI* and *FvCO* in six eastern and six western accessions. Leaf samples were collected at zeitgeber time 4 (ZT4) under 18-h long day (LD^{18h}) conditions and analysed using RNA sequencing. Error bars represent ±SD from 2 – 3 biological replicates (dots). (B) Pearson's correlations between relative expression of *FvFT1* in leaves (LD^{18h}, ZT16) and *FvTFL1* in shoot apices (LD^{18h}) and flowering time observations in a greenhouse after 12-h short day (SD^{12h}) treatment of six weeks or in plants induced to flower in the field in Helsinki in August-October. After treatments, flowering time observations were carried out in the greenhouse under LD^{18h}. The gene expression data is from Fig. 5h. (C) Pearson's correlation between relative expression of indicated genes. Leaf samples used in Fig. 5e were analyzed. *P*-values in B - C were estimated using Pearson's correlation test, Bonferroni adjusted. (D) Heatmap showing Pearson's correlations between flowering time and temperature related bioclimatic variables (bio1-bio11), longitude and latitude. Flowering time data from SD^{12h} treatment of five weeks (179 accessions), field experiment (181 accessions), and their average (179 accessions) were used. Asterisks mark the significance levels (***, $p < 0.001$; **, $p < 0.01$; *, $p < 0.05$, Bonferroni adjusted). Bioclimatic variables: BIO1 = Annual Mean Temperature; BIO2 = Mean Diurnal Range (Mean of monthly (max temp - min temp)); BIO3 = Isothermality (BIO2/BIO7) (×100); BIO4 = Temperature Seasonality (standard deviation ×100); BIO5 = Max Temperature of Warmest Month; BIO6 = Min Temperature of Coldest Month; BIO7 = Temperature Annual Range (BIO5-BIO6); BIO8 = Mean Temperature of Wettest Quarter; BIO9 = Mean Temperature of Driest Quarter; BIO10 = Mean Temperature of Warmest Quarter; BIO11 = Mean Temperature of Coldest Quarter.
